# Targeted anti-cancer therapy: Co-delivery of VEGF siRNA and Phenethyl isothiocyanate (PEITC) via cRGD-modified lipid nanoparticles for enhanced anti-angiogenic efficacy

**DOI:** 10.1016/j.ajps.2024.100891

**Published:** 2024-02-23

**Authors:** Bao Li, Haoran Niu, Xiaoyun Zhao, Xiaoyu Huang, Yu Ding, Ke Dang, Tianzhi Yang, Yongfeng Chen, Jizhuang Ma, Xiaohong Liu, Keda Zhang, Huichao Xie, Pingtian Ding

**Affiliations:** a School of Pharmacy, Shenyang Pharmaceutical University, Shenyang 110016, China; b School of Life Science and Biopharmaceutics Shenyang Pharmaceutical University Shenyang 110016, China; c Department of Basic Pharmaceutical Sciences School of Pharmacy Husson University Bangor, ME 04401, USA; d College of Pharmacy, Shenzhen Technology University, Shenzhen 518118, China

**Keywords:** Anti-angiogenesis, Tumor apoptosis, Nanoparticles, VEGF siRNA, Hypoxia inducible factor (HIF)-1 protein, Phenethyl isothiocyanate (PEITC)

## Abstract

Anti-tumor angiogenesis therapy, targeting the suppression of blood vessel growth in tumors, presents a potent approach in the battle against cancer. Traditional therapies have primarily concentrated on single-target techniques, with a specific emphasis on targeting the vascular endothelial growth factor, but have not reached ideal therapeutic efficacy. In response to this issue, our study introduced a novel nanoparticle system known as CS-siRNA/PEITC&L-cRGD NPs. These chitosan-based nanoparticles have been recognized for their excellent biocompatibility and ability to deliver genes. To enhance their targeted delivery capability, they were combined with a cyclic RGD peptide (cRGD). Targeted co-delivery of gene and chemotherapeutic agents was achieved through the use of a negatively charged lipid shell and cRGD, which possesses high affinity for integrin α_v_β_3_ overexpressed in tumor cells and neovasculature. In this multifaceted approach, co-delivery of VEGF siRNA and phenethyl isothiocyanate (PEITC) was employed to target both tumor vascular endothelial cells and tumor cells simultaneously. The co-delivery of VEGF siRNA and PEITC could achieve precise silencing of VEGF, inhibit the accumulation of HIF-1α under hypoxic conditions, and induce apoptosis in tumor cells. In summary, we have successfully developed a nanoparticle delivery platform that utilizes a dual mechanism of action of anti-tumor angiogenesis and pro-tumor apoptosis, which provides a robust and potent strategy for the delivery of anti-cancer therapeutics.

## Graphical abstract

**Figure:**
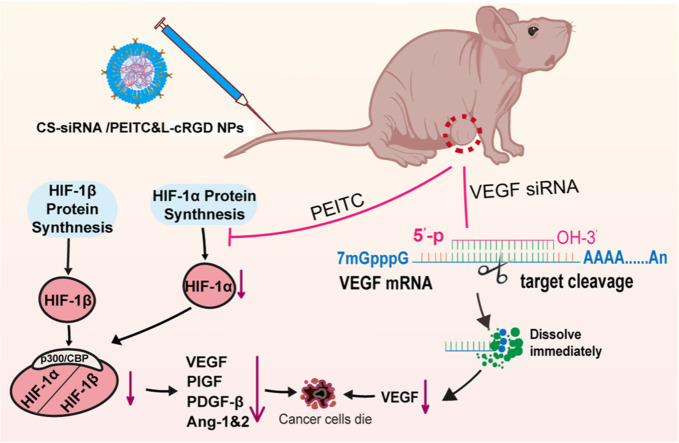

## 1 Introduction

Anti-angiogenesis has received considerable recognition as a therapeutic method for managing cancer, supplementing conventional modalities such as chemotherapy, radiotherapy, and surgery. This approach is based on the restriction of blood vessel formation in tumors, thus preventing their growth and metastasis [1]. This surge of interest is rooted in the crucial function that angiogenic mechanisms play in the advancement and spread of tumors. Tumor angiogenesis is the biological process in which new blood vessels form from pro-existing vasculature. The vascular network supplies vital nutrients to tumors while removing metabolic waste, thereby promoting tumor growth and facilitating metastasis [2,3]. Vascular endothelial growth factor (VEGF) is a glycoprotein demonstrating high biological activity, functioning as a potent endothelial cell mitogenic agent. It specializes in stimulating vascular endothelial cell proliferation, migration, and lumen formation [4], [5], [6], [7]. Angiogenesis inhibitors, which primarily target VEGF, have been developed. However, these single therapies have only shown efficacy in a limited number of cancers [1]. This limitation can be attributed to the intricacies of tumor angiogenesis and the involvement of hypoxia-inducible factor-1 (HIF-1), which acts as an upstream gene regulator with a pivotal role in the angiogenesis process [8], [9], [10]. HIF-1 is an integral part of tumor adaptation to hypoxic environments. Under low oxygen conditions, HIF-1α stabilizes and accumulates, forming a transcriptionally active complex by dimerization with HIF-1β [11], [12], [13], [14]. The active HIF-1 complex then binds to the hypoxia response element (HRE) in target genes, inducing VEGF expression [15]. Beyond VEGF, HIF-1 also regulates other angiogenic factors such as placenta-like growth factor, platelet-derived growth factor beta, and angiopoietin-1 and -2 [16].

VEGF small interfering RNA (siRNA) is a precise and efficient tool for silencing VEGF expression. By blocking the VEGF signaling pathway, it inhibits tumor angiogenesis [17,18]. Additionally, the specificity of siRNA allows it to identify its targeted mRNA, thus reducing the toxic side effects caused by off-targeting. After the target mRNA undergoes degradation, the RNA-induced silencing complex (RISC) can recycle, therefore increasing the silencing impact of siRNA [19], [20]. This enables continuous gene silencing with minimal amounts of siRNA molecules delivered into the cytoplasm. However, the delivery of siRNA drugs to target cells for clinical use presents significant challenges due to factors such as nuclease degradation and the hydrophilic and electronegative properties of siRNA that impede transmembrane and cellular uptake [21], [22], [23], [24]. Meanwhile, phenethyl isothiocyanate (PEITC), a compound found in cruciferous vegetables, has demonstrated a significant ability to protect against chemically induced cancer in animal trials [25]. Studies have confirmed that PEITC can inhibit cell proliferation in a dose-dependent manner, induce G2/M cell cycle arrest, deplete glutathione (GSH), generate reactive oxygen species (ROS), alter iron metabolism, and induce various forms of cell death [26]. PEITC effectively inhibits HIF-1α expression by suppressing the PI3K and MAPK pathways, preventing the accumulation of HIF-1α during hypoxia [27]. In addition, PEITC inhibits angiogenesis and migration by inactivating Akt, inhibiting pro-angiogenic growth factors, and reducing VEGF-R2 protein expression [28]. However, the hydrophobic nature of PEITC and its low bioavailability, coupled with safety concerns stemming from a narrow therapeutic index, require strategies for increasing its efficacy.

**Fig. 1:**
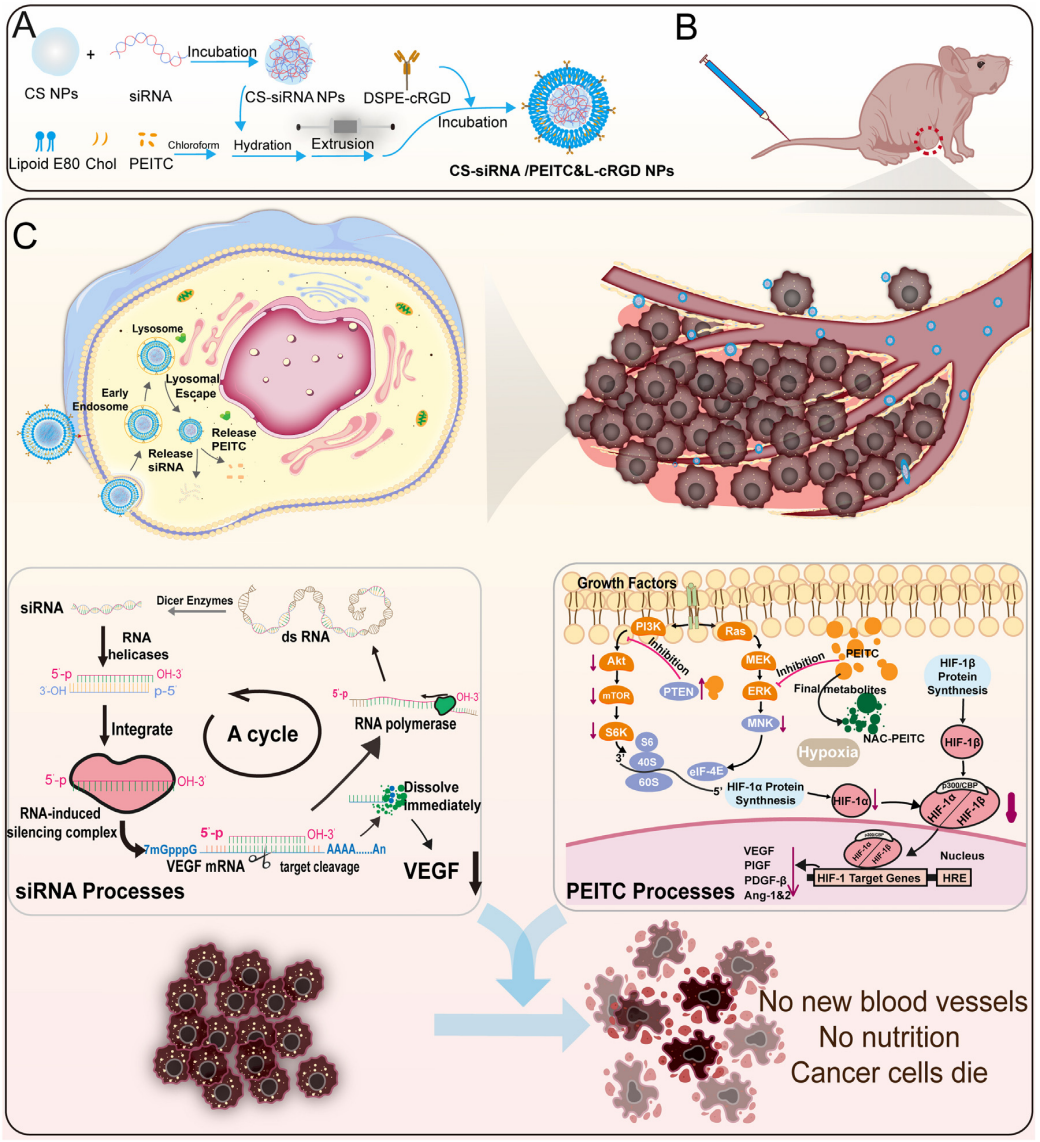
Schematic of the preparation and action mechanism of CS-siRNA/PEITC&L-cRGD NPs. (A) The process of CS-siRNA/PEITC&L-cRGD NPs synthesis. (B) The nanoparticles are introduced into mice via tail vein injection. (C) The mechanism of tumor angiogenesis inhibition by CS-siRNA/PEITC&L-cRGD NPs is depicted.

Given these challenges, we developed a co-delivery system using nanoparticles targeting tumor vasculature and cells to deliver siRNA and PEITC. This approach seeks to leverage the combined anti-angiogenic and pro-apoptotic capabilities to treat ectopic A549 solid tumors (Fig. 1). Our strategy involves the initial preparation of chitosan nanoparticles (CS NPs), which are subsequently loaded with siRNA (CS-siRNA NPs) by electrostatic adsorption. CS are widely used for gene delivery due to their excellent biosafety and degradability [29], [30], [31], [32]. Lipoid E 80, which is refined from egg yolk lecithin, was dissolved with cholesterol and PEITC in chloroform, and the resulting solution was used to prepare liposomes (PEITC&L) by the film dispersion method. The CS-siRNA NPs surface is coated with PEITC&L (CS-siRNA/PEITC&L NPs) utilizing a liposome extruder. This coating alters the charge of the NPs from positive to negative, leading to decreased cytotoxicity and enhanced blood circulation capabilities. The nanoparticles are modified with cRGD (CS-siRNA/PEITC&L-cRGD NPs), a peptide demonstrating high affinity for integrin α_v_β_3_, which is overexpressed in tumor cells and tumor vascular endothelial cells. This study provides a thorough and detailed account of the therapeutic effects achieved through the simultaneous delivery of siRNA and PEITC in the anti-angiogenic and pro-tumor apoptosis treatment.

## 2 Materials and methods

### 2.1 Materials

Chitosan (CS) with molecular weight of 100 kDa and deacetylation degree of 90% was obtained from Golden-Shell Biomedical Co., Ltd. (Zhejiang, China). PEITC, sodium tripolyphosphate (TPP), sodium acetate/acetic acid (pH4.0) buffer, phosphate-buffered saline (PBS), Tween 80 and cholesterol were obtained from SIGMA-ALDRICH (SHANGHAI) Trading Co., Ltd. (Shanghai, China). Lipoid E 80 was obtained from Lipoid GmbH (Ludwigshafen, Germany). VEGF siRNA (sense-GCCAGCACAUAGGAGAGAUTT, antisense-AUCUCUCCUAUGUGCUGGCTT) and Cy5-VEGF-siRNA were obtained from Shanghai Genepharma Co., Ltd. (Shanghai, China). 1,2-dipalmitoyl sn‑glycero-3-phosphoethanolamine-N-(polyethyleneglycol)-c(RGDyk) (DSPE-PEG2000-cRGD) was obtained from Xi'an Ruixi Biological Technology Co., Ltd. (Xi'an, China). CCK-8 assay kit, Annexin V-FITC/PI apoptosis detection kit, chlorpromazine, indomethacin, amiloride, β-cyclodextrin, Hoechst 33342, DiO, DAPI, VEGF antibody, HIF-1α antibody, and DiR enzyme-linked immunosorbent assay (ELISA) reagent for the detection of VEGF and ELISA reagent for the detection of HIF-1α were all obtained from Beyotime Institute of Biotechnology (Shanghai, China). Human umbilical vein endothelial cells (HUVEC) and human non-small cell lung cancer cells (A549) were procured from the Shanghai Institute of Cell Biology (Shanghai, China). Endothelial cell medium (ECM), Dulbecco's modified eagle medium (DMEM), trypsin, endothelial cell medium (ECM) and fetal bovine serum (FBS) were all purchased from Corning, Inc. (New York, USA). HE staining kit, VEGF-DAB staining kit, HIF-1α-DAB staining kit, TdT mediated dUTP nick end labeling (TUNEL) apoptosis detection kit (Alexa Fluor 640), and FITC anti-mouse CD31 antibody (FITC—CD31) were all purchased from Roche (Basel, Switzerland).

### 2.2 Preparation of CS-siRNA/PEITC&L-cRGD NPs

CS was dissolved in a sodium acetate/acetic acid buffer (0.2 mM, pH 4.0) at a concentration of 0.5 mg/ml and stirred magnetically at 150 rpm for 12 h at 25 °C. In parallel, TPP was dissolved in ultrapure water to achieve a concentration of 0.5 mg/ml. Both CS and TPP solutions were filtered through a 0.22 µm filter prior touse. CS NPs were subsequently synthesized by ionic crosslinking [33]. Chitosan nanoparticles loaded with siRNAs (CS-siRNA NPs) were prepared by adding TPP solution to a chitosan solution with a ratio of 1:5 (w/w), followed by centrifugation at 13,000 g for 30 min and redispersion in nuclease-free water. CS-siRNA NPs were obtained by incubating CS NPs with siRNA (CS to siRNA mass ratio of 10:1) for 30 min. SiRNA was absorbed onto the surface of CS NPs via electrostatic interaction.

For the PEITC&L preparation, Lipoid E 80, cholesterol, and PEITC were combined in a mass ratio of 10:2:1 and dissolved in chloroform. The solvent was subsequently evaporated under reduced pressure to form a homogeneous film on the flask's interior surface. Any residual chloroform was removed by nitrogen purging. The film was hydrated with 1 ml CS-siRNA NPs suspension (siRNA concentration of 100 µg/ml), incubated at 45 °C for 30 min, and then extruded seven times through a LiposoFast extruder fitted with a 200 nm polycarbonate membrane. This process wrapped the phospholipid film around the surface of the chitosan nanoparticles. Following the extrusion, the nanoparticle dispersion was centrifuged at 150 g for 3 min at a low temperature. The nanoparticle dispersion was retained to remove the uncoated PEITC precipitate, then the dispersion was centrifuged at 8000 g for 10 min, the supernatant containing free siRNA was discarded and redispersed in PBS. 0.1 mg DSPE-PEG2000-cRGD was added to the dispersion and incubated for 2 h with shaking, ensuring the DSPE-PEG2000-cRGD bound to the lipid membrane. The free cRGD was removed by centrifugation at 8000 g for 10 min, and the resulting precipitate was resuspended to yield the CS-siRNA/PEITC&L-cRGD NPs.

### 2.3 Characterization of CS-siRNA/PEITC&L-cRGD NPs

#### 2.3.1 Analysis of particle size, zeta potential and electron microscopy observations

Suitable volumes of deionized water dispersions of CS-siRNA NPs, PEITC&L, and CS-siRNA/PEITC&L-cRGD NPs were used to determine particle size and zeta potential using a Malvern particle size potentiometer (Malvern Instruments, UK). For electron microscopy observations, an appropriate volume of nanoparticle deionized water dispersion was applied dropwise onto the copper mesh, stained with uranyl acetate, and the microscopic morphology of CS-siRNA NPs, PEITC&L, and CS-siRNA/PEITC&L-cRGD NPs were visualized at 100 kV by transmission electron microscopy (JEM 1200EX, JEOL, Japan).

#### 2.3.2 Assessment of siRNA encapsulation capacity

The encapsulation capacity of co-delivered nanoparticles for siRNA was evaluated using agarose gel electrophoresis. Both CS-siRNA NPs and CS-siRNA/PEITC&L-cRGD NPs were mixed with the loading buffer to achieve a final siRNA concentration of 100 µg/ml. The samples were then loaded into the wells of the agarose gel and subjected to electrophoresis for 30 min at a steady voltage of 110 V. Free siRNA was utilized as a negative control. Following electrophoresis, gel images were captured using a gel imaging system (1600, Tanon, China).

#### 2.3.3 Determination of encapsulation rate and drug loading capacity

To determine the drug loading capacity and encapsulation rate, CS-Cy5-siRNA/PEITC&L-cRGD NPs were prepared by modifying siRNA with the fluorescent dye Cy5. The nanoparticles were then weighed after lyophilization of their aqueous dispersion to measure their mass. To determine the free drug content, the supernatant contained free Cy5-siRNA, and the precipitate contained PEITC, both of which were collected during the nanoparticle preparation process. The concentration of unbound Cy5-siRNA was evaluated through fluorometric spectrophotometry (Excitation/Emission (Ex/Em): 650 nm/670 nm), whereas unbound PEITC was measured via UV–Visible spectrophotometry at an absorbance of 251 nm. The encapsulation efficiency (EE) and drug loading (DL) were computed using Eqs. (1) and (2).

(1) $$\text{EE}\;(\%)=\frac{m_{Initial\;drug\;content\;}-m_{Free\;drug\;content}}{m_{Initial\;drug\;content\;}}\times 100\%$$

(2) $$DL\;\left(\%\right)=\frac{m_{Initial\;Drug\;Content}-m_{Free\;Drug\;Content}}{m_{\;Nanoparticles}}\times 100\%$$

#### 2.3.4 Serum stability assessment

The *in vitro* stability of CS-siRNA/PEITC&L-cRGD NPs was assessed using a serum stability assay. In this procedure, CS-siRNA/PEITC&L-cRGD NPs were incubated with 50% fetal bovine serum (FBS, v/v) at 37 °C across a range of concentrations (corresponding to PEITC concentrations: 1, 5, 20, 100 and 200 µg/ml). The absorbance was then measured at several time points (0, 1, 2, 4, 6, 8, 12, 24 and 48 h) using a multifunctional enzyme marker (Spectra Max M3, Molecular Devices, USA) at an absorbance of 560 nm. An equal volume of PBS was utilized as a blank control. Six replicate samples were prepared for each time point. Relative turbidity was calculated as in Eq. (3).

(3) $$Relative\;turbidity=\;\frac{A_{Each\;time\;point}}{A_{Time\;0}}$$

#### 2.3.5 Hemolysis evaluation

The biocompatibility of CS-siRNA/PEITC&L-cRGD NPs with erythrocytes was evaluated by examining the hemolysis. Erythrocytes were extracted from the whole blood of BALB/c nude mice and rinsed with cold, sterile saline until the supernatant was clear. A 300 µl erythrocyte suspension was thoroughly mixed with gradient concentrations of CS-siRNA/PEITC&L-cRGD NPs (200 µl) in Eppendorf (EP) tubes, corresponding to PEITC concentrations of 1, 5, 20, 100 and 200 µg/ml. The negative control group consisted of PBS (resulting in 0 hemolysis), while the positive control group used deionized water (resulting in 100% hemolysis). After 12 h of incubation at 37 °C, the samples were centrifuged at 720 g for 15 min, and then photographed to document the condition of the samples inside the EP tubes. Absorbance was measured using a multifunctional enzyme marker at 540 nm with three replicates per group. The hemolysis rate (%) was calculated as in Eq. (4).

(4) $$Hemolysis\;\left(\%\right)=\frac{A_{Sample}-A_{PBS}}{A_{Deionized\;water}-A_{PBS}}\times 100\%$$

#### 2.3.6 Evaluation of *in vitro* release

To study the siRNA and PEITC release from CS-siRNA/PEITC&L-cRGD NPs, the sample of CS-siRNA/PEITC&L-cRGD NPs (at a PEITC concentration of 10 mg/ml, and the Cy5-siRNA concentration of 1 mg/ml) was placed in dialysis bags (molecular weight cut-off: 30 kDa), which were in turn immersed in simulated physical environment (pH 7.4 PBS containing 0.1% Tween 80), and endosome (0.5 mg/ml lysozyme in PBS at pH 5.0). At specified time points (0, 0.5, 1, 2, 4, 6, 8, 12, 24 and 48 h), samples were withdrawn and replaced with equal volumes of fresh dialysis medium. Three replicate samples were maintained for each time point. The concentration of Cy5-siRNA was evaluated through fluorometric spectrophotometry (Ex/Em: 650 nm/670 nm), whereas PEITC was measured via UV–Visible spectrophotometry at an absorbance of 251 nm, and the results were used to analyze the cumulative release rate.

### 2.4 *In vitro* biological evaluation of CS-siRNA/PEITC&L-cRGD NPs

#### 2.4.1 Cell culture

HUVEC cells were cultured in ECM and A549 cells were cultured in DMEM. Cells were cultured under standard experimental conditions in a humidified incubator with 5% CO_2_ at 37 °C. All cells were cultured and collected according to standard experimental protocols.

#### 2.4.2 Cytotoxicity evaluation

The cytotoxicity of CS-siRNA/PEITC&L-cRGD nanoparticles on HUVEC and A549 cells was evaluated using the CCK-8 method as instructed. Briefly, HUVEC and A549 cells were inoculated into 96-well plates at a density of 5 × 10³ per well, respectively, and cultured overnight. The cells were co-incubated with free siRNA, CS-siRNA NPs, and CS-siRNA/L-cRGD NPs using concentrations that corresponded to final siRNA concentrations of 0, 10, 20, 30, 50, 80 and 100 nM. Similarly, the cells were co-incubated with free PEITC, PEITC&L, and CS-siRNA/PEITC&L-cRGD NPs at concentrations equivalent to the final PEITC concentrations of 0, 1, 2, 5, 10, 20, 30 and 50 µM. Following a 24 h incubation at 37 °C, cells were processed as per the kit's standard procedure. Absorbance was measured at 450 nm using a multifunctional enzyme marker. Cell viability was then calculated as in Eq. (5).

(5) $$Cell\;viability\;\left(\%\right)=\frac{A_{Expermental\;group}-A_{Blank\;group}}{A_{Control\;group}-A_{Blank\;group}}\times 100\%$$

#### 2.4.3 Cell apoptosis analysis

The induction of apoptosis by CS-siRNA/PEITC&L-cRGD NPs was evaluated using the Annexin V-FITC/PI apoptosis detection kit. HUVEC and A549 cells were separately seeded in 6-well plates at a density of 3 × 10⁵ cells per well, respectively, and cultured overnight. The cells were treated with free siRNA, free PEITC, CS-siRNA/L-cRGD NPs, PEITC&L-cRGD, and CS-siRNA/PEITC&L-cRGD NPs at a concentration equivalent to 30 µM PEITC and 100 nM siRNA, followed by incubation at 37 °C for 24 h. An equal volume of PBS served as the blank control group. After incubation, the cells were trypsinized, collected by centrifugation, and then resuspended in PBS to a density of 1 × 10⁶ cells/ml. A 100 µl aliquot of binding buffer was added to the suspension along with 5 µl each of Annexin V-FITC (Ex/Em: 495 nm/520 nm) and propidium iodide (Ex/Em: 493 nm/636 nm). The samples were then incubated for 20 min at room temperature in the dark. The extent of apoptosis was analyzed by flow cytometry (BD FACSAria II, MD, USA).

#### 2.4.4 Cellular uptake analysis

The cellular uptake of CS-siRNA/PEITC&L-cRGD NPs was evaluated using HUVEC and A549 cells. Both cell types (at a density of 3 × 10⁵ cells per well) were separately seeded into 6-well plates, respectively, and cultured overnight. The cells were then treated with Free siRNA, CS-siRNA/PEITC&L NPs, and CS-siRNA/PEITC&L-cRGD NPs (Fluorescently labeled siRNA with Cy5), PBS served as a negative control. All treatments were applied at an equal siRNA concentration of 5 µg/ml. After 4 h of incubation, the cells were washed thoroughly with PBS, detached using trypsin, collected by centrifugation, and then resuspended in PBS. The degree of cellular uptake was then assessed by flow cytometry (BD FACS Aria II, MD, USA).

#### 2.4.5 Evaluation of endocytic mechanism

The mechanism of endocytic uptake for CS-siRNA/PEITC&L-cRGD nanoparticles was investigated using HUVEC and A549 cells. These cells (at a density of 3 × 10⁵ cells per well) were cultured in 6-well plates overnight. The cells were then pre-treated for 1 h with chlorpromazine (10 µg/ml), indomethacin (8 µg/ml), amiloride (5 µg/ml), and β-cyclodextrin (8 µg/ml), respectively. Following pre-treatment, the cells were exposed to CS-siRNA/PEITC&L-cRGD nanoparticles at a concentration of 5 µg/ml of siRNA. After a co-incubation period of 12 h, the cells were washed thoroughly with PBS, detached using trypsin, collected by centrifugation, and then resuspended in PBS. The uptake mechanism of the CS-siRNA/PEITC&L-cRGD NPs was subsequently analyzed using flow cytometry (mean fluorescence intensity, MFI). The uptake inhibition rate (%) was then calculated as in Eq. (6).

(6) $$\begin{array}{ccc} & & Uptake\;Inhibition\;Rate\;\left(\%\right)\\ & & =\frac{MFI_{Control\;group}-MFI_{Experimental\;group}}{\;MFI_{Control\;group}}\times 100\% \end{array}$$

#### 2.4.6 Confocal laser scanning microscopy (CLSM) analysis

The intracellular distribution of CS-siRNA/PEITC&L-cRGD NPs was evaluated using HUVEC and A549 cells. Both cell types (at a density of 1 × 10⁵ cells per well) were seeded into 12-well plates containing coverslips and cultured overnight. Cells were then co-incubated with Free siRNA, CS-siRNA/PEITC&L NPs, and CS-siRNA/PEITC&L-cRGD NPs (Fluorescently labeled siRNA with Cy5), PBS served as a negative control, while LIPO2000-Cy5-siRNA was used as a positive control. The final concentration for each treatment was equivalent to the siRNA concentration: 5 µg/ml. After a 4-h co-incubation period, the medium was discarded, and the cells were washed three times with PBS. Cells were then fixed with a 4% paraformaldehyde solution for 20 min at 25 °C. Staining solutions containing Hoechst 33,342 (for nuclear staining) and DiO (for staining the cell membrane) were added in sequence, with each solution followed by a rinse with PBS. After washing, the coverslips were mounted onto slides to prepare them for viewing. The intracellular distribution of nanoparticles was visualized using a laser scanning confocal microscope (LSCM, LSM710, Carl Zeiss, Germany) operating in three-channel mode (green channel = DiO: λex: 480 nm, λem: 520 nm; blue channel = Hoechst 33342: λex: 346 nm, λem: 460 nm; red channel = Cy5: λex: 650 nm, λem: 670 nm).

### 2.5 *In vivo* biological evaluation of CS-siRNA/PEITC&L-cRGD NPs

#### 2.5.1 Animal care and tumor model establishment

All animals used in this study were obtained from the Experimental Animal Center of Shenyang Pharmaceutical University (Shenyang, Liaoning, China). The experimental procedures were reviewed and approved by the Animal Care and Use Committee of Shenyang Pharmaceutical University (SPYU-IACUC—C2018–10–10–87). To establish the BALB/c nude ectopic tumor model, A549 cells (2 × 10⁶ cells per mouse) were subcutaneously injected into the axillary region of the right forelimb of female BALB/c nude mice (approximately 5–6 weeks old). Tumor growth was monitored daily using calipers, and tumor volume was calculated as in Eq. (7). In accordance with the institutional policies, mice were humanely euthanized if the tumor volume exceeded 2,000 mm³ or if the tumor size surpassed 20 mm in any dimension.

(7) $$Volume\;\left(mm^{3}\right)=\frac{Length\times Width^{2}}{2}$$

#### 2.5.2 *In vivo* tumor-targeted accumulation evaluation

The efficiency of tumor targeting and accumulation of CS-siRNA/PEITC&L-cRGD NPs *in vivo* were evaluated through the utilization of Dir-labeled (Ex/Em: 720 nm/790 nm) preparations for *in vivo* imaging. When the average tumor volume reached 400 mm³, BALB/c nude mice were administered CS-siRNA/PEITC&L-cRGD NPs and CS-siRNA/PEITC&L NPs (dosage: PEITC 10 mg/kg and siRNA 1 mg/kg body weight), respectively, via tail vein injection. During the imaging process, mice were anesthetized with 1.25% isoflurane. Using an *in vivo* imaging system (IVIS, Caliper Life Science, MA, USA), anesthetized mice were imaged, and the fluorescent signal in the tumor region was analyzed using Living Image 4.0 software at 1, 2, 4, 6, 12 and 24 h post-injection. After the final *in vivo* imaging session, mice were euthanized and vital organs (including the liver, spleen, kidney, heart, and lungs) along with tumors were harvested for *ex vivo* imaging. BALB/c nude mice with tumors, but no other treatments, were utilized as control groups to eliminate background fluorescence.

#### 2.5.3 Evaluation of tumor vascular targeting

To assess the ability of CS-siRNA/PEITC&L-cRGD NPs to specifically target tumor vasculature and tumor cells, a co-localization analysis was carried out. Following ex vivo imaging, the isolated tumors underwent fixation in 4% paraformaldehyde, embedding in paraffin, and sectioning into 5 µm slices. The nuclei of the tumor cells were stained with DAPI, whereas the blood vessels in the tumor were labeled using FITC—CD31. Fluorescent images of the stained sections were captured and recorded utilizing a panoramic section scanner. (Panorama P250; 3D HISTECH, Budapest, Hungary).

#### 2.5.4 *In vivo* antitumor efficacy evaluation

The A549 tumor model, established as previously mentioned, was utilized to assess the efficacy of antitumor treatment *in vivo*. Upon reaching a mean tumor volume of 350–450 mm³, the mice bearing the tumor were randomly assigned to groups (*n* = 5 per group). The intravenous treatment regimens administered were as follows: PBS, free PEITC, CS-siRNA/PEITC&L NPs, CS-siRNA/L-cRGD NPs, PEITC&L-cRGD, and CS-siRNA/PEITC&L-cRGD NPs. Final concentrations for these treatments were 10 mg/kg body weight for PEITC and 1 mg/kg body weight for siRNA. Treatments were given at 2-d intervals, for a total of 10 treatments. The body weights and tumor volumes of the mice were continuously monitored and recorded during the treatment period. Following the final recording, the mice were euthanized. Standard procedures were used to conduct histochemical and immunohistochemical staining of isolated tumors and organs. CD31-DAB staining was used to observe the distribution of blood vessels in the tumor's central area. VEGF-DAB staining allowed for the observation of VEGF expression levels in the tumor's central area, while HIF-1α-DAB staining was used to assess the expression levels of HIF-1α in the same area. To observe necrosis and apoptosis in the central and marginal areas of the tumor, TUNEL-DAB staining was performed. Semi-quantitative or quantitative analysis was conducted using NIH ImageJ software (NIH, Bethesda, MD, USA). In brief, after loading the image, the appropriate area for immunostaining was marked, the intensity of the immunostaining was calculated using the measure tool, and the area and density of the immunostaining were analyzed using the analyze particles tool.

#### 2.5.5 Evaluation through ELISA and Western blot

The expression levels of HIF-1α and VEGF were quantified in tumor tissues using both enzyme-linked immunosorbent assay (ELISA) and Western Blot techniques across all groups. The tumor tissues were thoroughly lysed (with three parallel samples for each group), and the total protein content for each group was determined by using a BCA protein quantification kit. Levels of HIF-1α and VEGF were quantified as per the instructions of the ELISA kit. For Western Blot analysis, proteins were separated by sodium dodecyl sulfate-polyacrylamide gel electrophoresis (SDS-PAGE) and then electrophoretically transferred to polyvinylidene difluoride membranes. The membrane were sealed by incubation with 5% skim milk for 1 h. The membranes were then incubated with primary antibodies (dilution as per manufacturer's instructions) overnight at 4 °C, followed by incubation with horseradish peroxidase-linked anti-rabbit or anti-mouse IgG as a secondary antibody. Ultimately, the PVDF film was treated with an ECL-illuminating solution followed by exposure, development, and fixing in a dark room for the purpose of analyzing HIF-1α and VEGF expressions.

#### 2.5.6 Safety evaluation

After the antitumor evaluation period was finished, we examined the major organs (heart, liver, spleen, lungs, and kidneys) for potential toxicity by conducting a histopathological analysis using hematoxylin and eosin (HE) staining. To further evaluate the tolerability and potential toxicity of high-dose intravenous administration, five healthy BALB/c nude mice were administered with CS-siRNA/PEITC&L-cRGD NPs (PEITC: 30 mg/kg body weight; siRNA: 3 mg/kg body weight) via the tail vein. A control group consisted of five mice that were administered an equivalent amount of PBS. After a 21-d observation period, all mice were euthanized and their major organs were extracted for histological analysis.

### 2.6 Statistical analysis

The quantitative data in the study were analyzed by GraphPad Prism 8 (San Diego, California USA), and presented as the mean ± standard deviation (SD). Statistical comparisons were made by unpaired Student's *t*-test (between two groups) and one-way ANOVA (for multiple comparisons). For quantitative analysis in HE, TUNEL, CD31-DAB, VEGF-DAB, HIF-DAB, and fluorescence confocal images, Image J software (National Institutes of Health, USA) was used for densitometric analysis.

## 3 Results and discussions

### 3.1 Preparation and characterization of CS-siRNA/PEITC&L-cRGD NPs

The construction of the co-delivery system, CS-siRNA/PEITC&L-cRGD NPs, consists of two components: a rigid core formed by chitosan nanoparticles loaded with siRNA (CS-siRNA NPs) and a lipid membrane shell carrying PEITC (PEITC&L). First, the positively charged chitosan nanoparticles, CS NPs, were prepared by ionic crosslinking method, followed by the generation of CS-siRNA NPs by co-incubation with negatively charged siRNA. Due to the strong electrostatic attraction between CS NPs and siRNAs, CS NPs can load siRNAs stably and efficiently. Subsequently, PEITC&L was prepared by the film dispersion method, and CS-siRNA NPs were used as the hydration solution to generate CS-siRNA/PEITC&L NPs. Using a LiposoFast extruder equipped with a 200 nm polycarbonate film, the flexible structure of phospholipids was wrapped around the surface of the rigid chitosan nanoparticle by a physical extrusion process. After modification with cRGD, CS-siRNA/PEITC&L-cRGD NPs were successfully obtained.

Effective control of the particle size of CS-siRNA NPs and the stability of the loaded siRNAs are critical to the stability of the core of the co-delivery system. The CS-siRNA NPs, which serve as the core, are the main factors that influence the particle size of the CS-siRNA/PEITC&L-cRGD NPs. The particle size of CS NPs was controlled by optimizing the crosslinking reaction variables of CS and TPP. The primary reaction variables include mass ratio, stirring speed, and stirring duration. By optimizing the co-incubation mass ratio of CS NPs with siRNA, we determined the optimal mass ratio for efficient and stable loading of siRNA onto CS NPs. According to our preliminary experiments, we were able to achieve the ideal particle size range of CS NPs under the conditions of CS to TPP mass ratio of 5:1, and magnetic stirring speed of 400 rpm for 30 min. In addition, controlling the co-incubation mass ratio of CS NPs to siRNAs to more than 10:1 enabled efficient and stable loading of siRNAs onto CS NPs.

The ability of PEITC&L to act as a surface film coating for co-delivery systems and to efficiently and stably encapsulate the core is critical to maintaining the integrity of the nanoparticle. The design of PEITC&L was aimed at achieving complete encapsulation of CS-siRNA NPs through optimization of the mass ratio of PEITC&L to CS-siRNA NPs. In addition to providing a protective barrier for CS-siRNA NPs, PEITC&L provides a modification site for cRGD. DSPE-PEG2000-cRGD was modified on the surface of CS-siRNA/PEITC&L NPs by exploiting the affinity of DSPE for phospholipid bilayers. Preliminary experiments showed that at a mass ratio of 1:1 between PEITC&L and CS-siRNA NPs, the system particles were positively charged, indicating that only adsorption aggregates could form at this ratio. However, when the mass ratio of PEITC&L to CS-siRNA NPs was increased to 2:1, the surface charge became negative, indicating that PEITC&L had achieved complete encapsulation of the core.

**Fig. 2:**
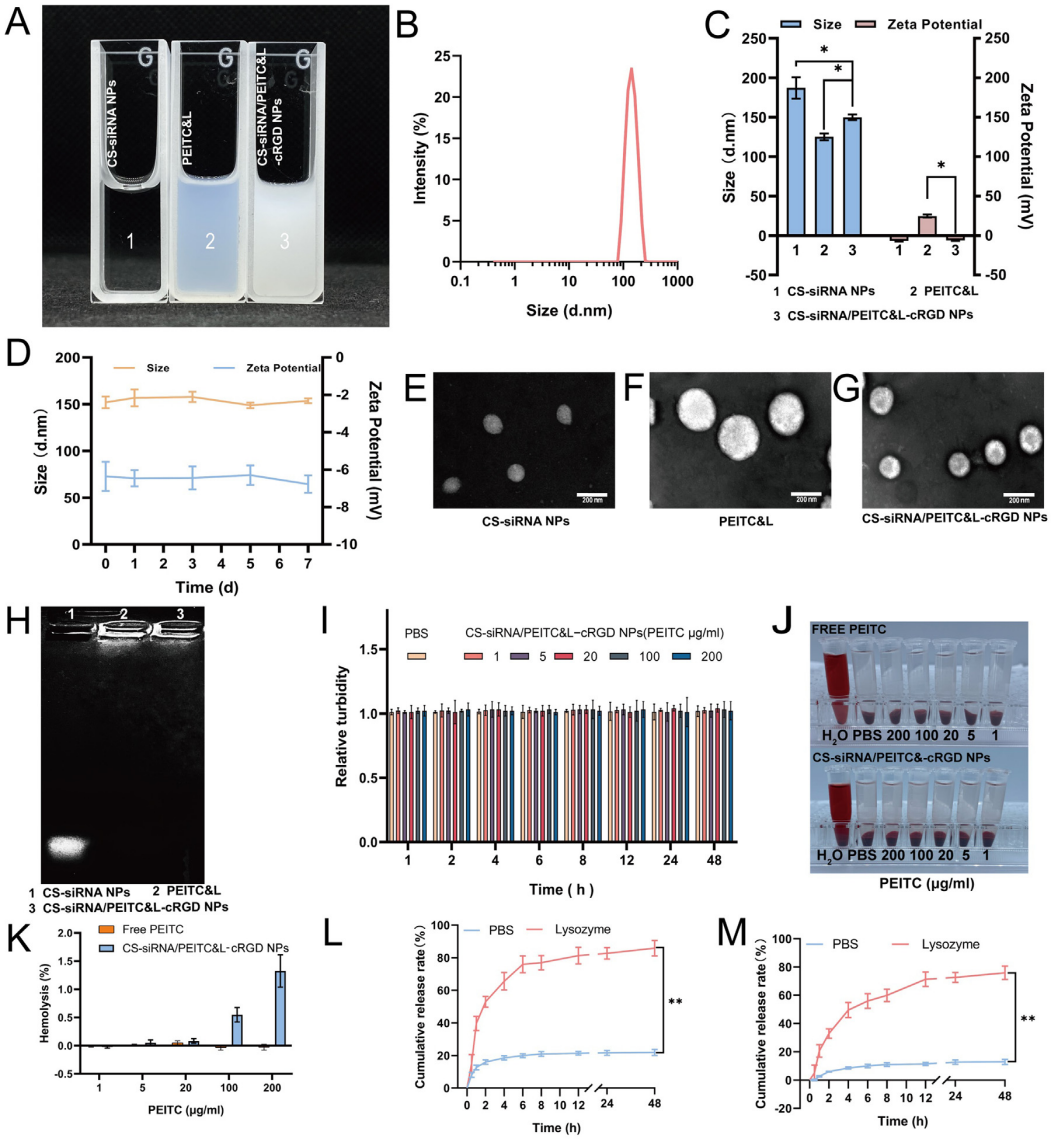
Characterization of CS-siRNA/PEITC&L-cRGD NPs. (A) Representative images showcasing the appearance of various formulations. (Label 1: CS-siRNA NPs, 2: PEITC&L, 3: CS-siRNA/PEITC&L-cRGD NPs). (B) Particle-size distribution of CS-siRNA/PEITC&L-cRGD NPs, analyzed by intensity (*n* = 3). (C) Comparative analysis of size and zeta potential for PEITC&L, CS-siRNA NPs and CS-siRNA/PEITC&L-cRGD NPs (Label 1: PEITC&L, 2: CS-siRNA NPs, 3: CS-siRNA/PEITC&L-cRGD NPs, *n* = 3). (D) Colloidal stability analysis of CS-siRNA/PEITC&L-cRGD NPs (*n* = 3). (E-G) TEM images of CS-siRNA NPs (E), PEITC&L (F) and CS-siRNA/PEITC&L-cRGD NPs (G). (H) The association ability of CS NPs with siRNA, evaluated before and after the addition of lipids to the complexes (Lane 1: Free siRNA, Lane 2: Before the addition of lipids, Lane 3: After the addition of lipids). (I) Serum stability analysis of PBS and CS-siRNA/PEITC&L-cRGD NPs (*n* = 5). (J) Hemolysis photographs of PBS and CS-siRNA/PEITC&L-cRGD NPs. (K) Analysis of the hemolysis rate (*n* = 3). (L) Cumulative release rate of PEITC (*n* = 3). (M) Cumulative release rate of siRNA (*n* = 3). Statistical significance is indicated as *P < 0.05, **P < 0.01, and ***P < 0.001.

Fig. 2A shows the appearance of CS-siRNA NPs, PEITC&L, and CS-siRNA/PEITC&L-cRGD NPs. As determined by dynamic light scattering (DLS), the average particle size of CS-siRNA/PEITC&L-cRGD NPs was 149.9 nm ± 4.9 nm (Fig. 2B-2C). The mean particle sizes of CS-siRNA NPs and PEITC&L were 125.2 nm ± 5.8 nm and 187.2 nm ± 12.5 nm, respectively. The average zeta potentials were +24.81 mV ± 2.1 mV for CS-siRNA NPs, −7.21 mV ± 2.7 mV for PEITC&L, and −5.91 mV ± 1.6 mV for CS-siRNA/PEITC&L-cRGD NPs (Fig. 2C). The DLS results indicated the successful encapsulation of CS-siRNA NPs by PEITC&L, resulting in the reversal of the positive charge on the surface of CS-siRNA NPs. The results of colloidal stability experiments (Fig. 2D) showed that CS-siRNA/PEITC&L-cRGD NPs exhibited excellent colloidal stability based on particle size, zeta potential and polydispersity index (PDI, all values below 0.2, data not shown) even after 7 d of storage. Transmission electron microscopy (TEM) results showed that the CS-siRNA NPs were monodisperse spheres (Fig. 2E) with a particle size of approximately 130 nm, similar to the DLS results. The TEM image of PEITC&L showed a particle size of 200 nm, similar to the DLS results (Fig. 2F). The CS-siRNA/PEITC&L-cRGD NPs exhibited a core-shell spherical structure (Fig. 2G) with a particle size of approximately 160 nm, consistent with the DLS-determined particle size. Furthermore, agarose gel electrophoresis results (Fig. 2H) showed that CS NPs could stably load siRNA, and PEITC&L encapsulation of CS-siRNA NPs did not affect the binding stability of CS NPs to siRNA.

The encapsulation rate of PEITC and siRNA was found to be 87.35% ± 1.79% and 98.85% ± 0.95%, respectively. The high encapsulation rate of both substances results from PEITC being loaded into the phospholipid bilayer and the efficient, stable binding of siRNA to CS NPs. Furthermore, the efficient encapsulation by siRNA also confirmed that siRNA and CS NPs could maintain stable binding during the physical extrusion process, and the structure of CS-siRNA NPs remained intact. DL of PEITC and siRNA were 6.03% ± 0.61% and 0.61% ± 0.05%, respectively. Based on the serum stability and hemolysis rate results (Fig. 2I-K), the CS-siRNA/PEITC&L-cRGD NPs displayed notable serum stability and minimal hemolysis within the concentration range of 0.1–200 µg/l for PEITC. These results provide preliminary evidence supporting the suitability of intravenously administering CS-siRNA/PEITC&L-cRGD NPs. The results of the *in vitro* drug release study demonstrated that 21.85% ± 1.75% release of PEITC (Fig. 2L) and 12.86% ± 1.76% of siRNA (Fig. 2M) after 48 h in PBS. In contrast, 85.85% ± 5.75% release of PEITC (Fig. 2L) and 75.16% ± 4.51% after 48 h in endosomal environment. Therefore, the CS-siRNA/PEITC&L-cRGD NPs maintained favorable integrity and demonstrated minimal PEITC and siRNA release over 48 h in PBS, establishing a basis for subsequent targeted tumor accumulation therapy.

### 3.2 *In vitro* biological evaluation of CS-siRNA/PEITC&L-cRGD NPs

#### 3.2.1 Cytotoxicity evaluation

**Fig. 3:**
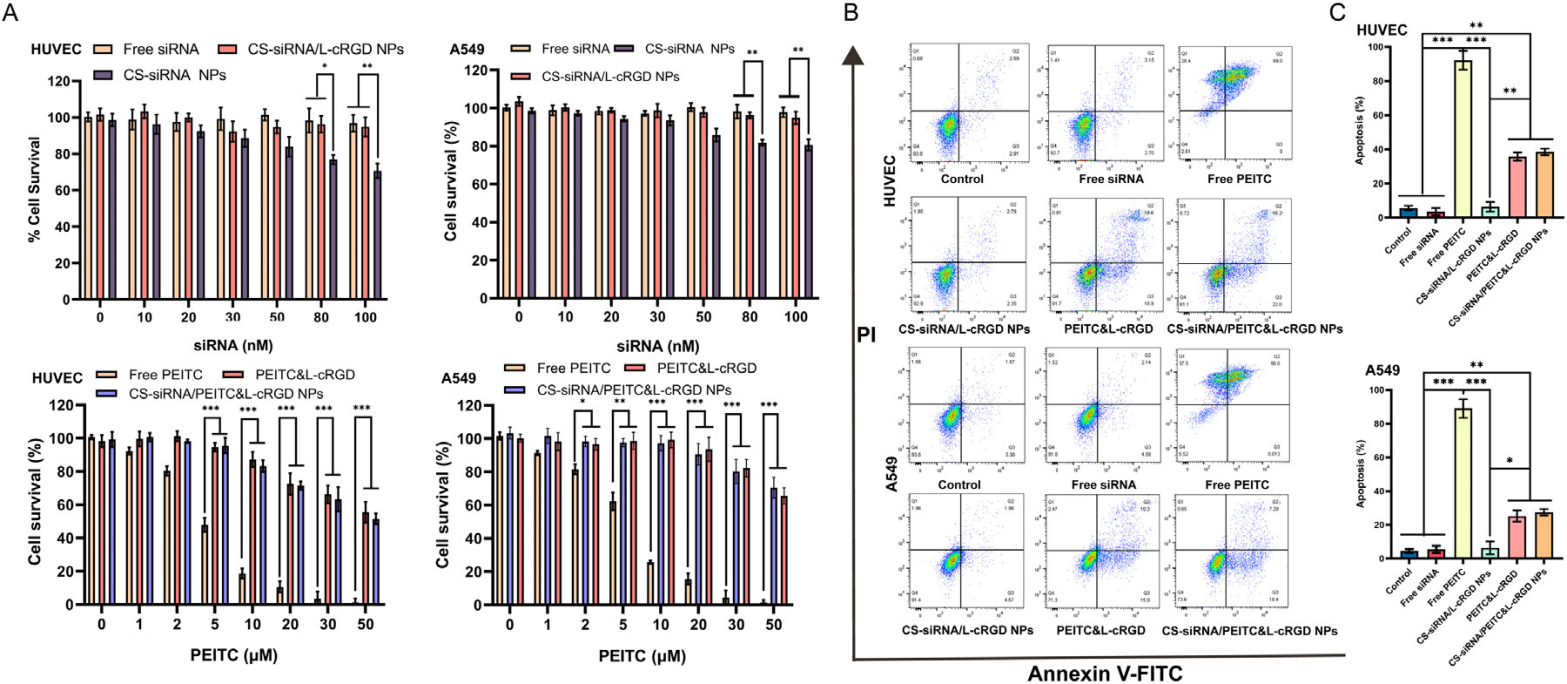
(A) Assessment of cytotoxic effects on HUVEC and A549 cells after 24 h exposure to various treatments: Free siRNA, CS-siRNA NPs, CS-siRNA/L NPs, Free PEITC, and CS-siRNA/PEITC&L-cRGD NPs (*n* = 6). (B) Scatter plots depicting the apoptosis rate in A549 and HUVEC cells after treatment with PBS, Free siRNA, Free PEITC, CS-siRNA/L-cRGD NPs, PEITC/L-cRGD, and CS-siRNA/PEITC&L-cRGD NPs. (C) Accompanied by an analysis of the apoptosis rate for both cell types (*n* = 3). Statistical significance is denoted as *P < 0.05, **P < 0.01, and ***P < 0.001.

As shown in Fig. 3A, Free siRNAs had no significant effect on either HUVEC or A549 cells. However, the cell survival of CS-siRNA NPs was significant for both cell types at siRNA concentrations of up to 80 nM (**P < 0.05). When the CS-siRNA NPs were coated with phospholipids (forming CS-siRNA/L-cRGD NPs), the cell survival was significantly reduced (**P < 0.05). This increase in survival is attributed to the change in surface charge from positive to negative brought about by the phospholipid coating. PEITC exhibited a concentration-dependent cell survival effect on HUVEC and A549 cells. When the PEITC concentration reached 5 µM, free PEITC demonstrated a significant survival effect on both HUVEC cells (***P < 0.001) and A549 cells (**P < 0.01). The survival rate of CS-siRNA/PEITC&L-cRGD NPs and PEITC&L-cRGD group was significantly higher than that of the free PEITC group due to the efficient shielding of the cytotoxic effect of PEITC by the phospholipid bilayer. This shielding effect enhanced the safety of the formulation in circulation. In addition, the IC-50 of CS-siRNA/PEITC&L-cRGD NPs against HUVEC and A549 cells was 87.19 ± 6.7 µM and 114 ± 5.3 µM, respectively. The results of the IC50 demonstrated that CS-siRNA/PEITC&L-cRGD NPs possess potent inhibitory effects on the viability of both A549 and HUVEC cells. This serves as a foundation for the anti-tumor efficacy *in vivo*.

#### 3.2.2 Apoptosis evaluation

The pro-apoptotic effect of PEITC has been reported in numerous studies, with the mechanism of action primarily involving cell cycle arrest and ROS production. The pro-apoptotic effect of CS-siRNA/PEITC&L-cRGD NPs was evaluated by Annexin V-FITC and PI staining. As shown in Fig. 3B and C, the apoptosis rates induced by CS-siRNA/PEITC&L-cRGD NPs in HUVEC cells and A549 cells were 38.45% ± 3.79% (**P < 0.01) and 25.11% ± 1.74% (**P < 0.01), respectively. Free siRNA and CS-siRNA/L NPs had no significant apoptotic effect on A549 cells and HUVEC cells. The apoptosis rate in the Free PEITC group (92.13% ± 4.39% in HUVEC, 88.31% ± 5.14% in A549) was significantly higher than the other groups due to cytotoxicity exacerbated apoptosis and necrosis in HUVEC and A549 cells. The apoptosis rate induced by PEITC&L-cRGD was similar to the results of CS-siRNA/PEITC&L-cRGD NPs, indicating that PEITC is a major pro-apoptotic factor. The CS-siRNA/PEITC&L-cRGD NPs exhibited pro-apoptotic effects on both cell types, with a stronger effect observed in HUVEC cells. The pro-apoptotic effect of CS-siRNA/PEITC&L-cRGD NPs was more effective in HUVEC cells compared to A549 cells. This difference may be due to A549 cells, being tumor cells, having a higher proliferation rate and increased viability than HUVEC cells.

#### 3.2.3 Cellular uptake

**Fig. 4:**
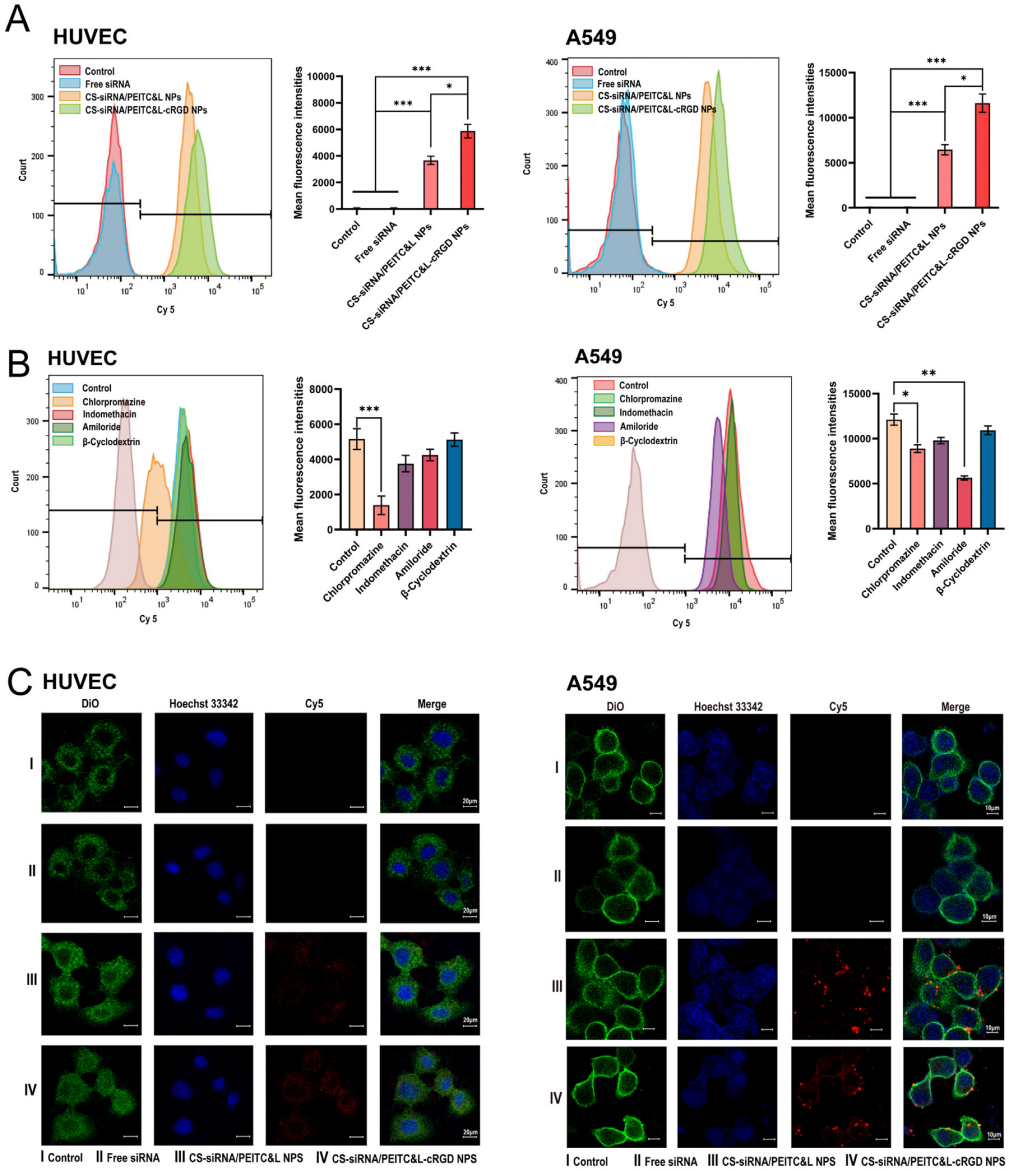
(A) Histogram representing the fluorescence intensity of Free siRNA, CS-siRNA/PEITC&L NPs, and CS-siRNA/PEITC&L-cRGD NPs in HUVEC and A549 cells. The graph also includes an analysis of the fluorescence intensity of HUVEC and A549 cells (*n* = 3). (B) Flow cytometric analysis depicting the inhibition of endocytic uptake in HUVEC and A549 cells (*n* = 3), along with an analysis of fluorescence intensity in HUVEC and A549 cells (*n* = 3). (C) CLSM images of HUVEC and A549 cells after 4 h incubation with Free siRNA, CS-siRNA/PEITC&L NPs, and CS-siRNA/PEITC&L-cRGD NPs. Cell nuclei and cell membranes were stained with Hoechst 33342 (blue) and DiO (green), respectively. Statistical significance is indicated by *P < 0.05, **P < 0.01, and ***P < 0.001.

SiRNA was modified with a fluorescent Cy5 tag to detect cellular uptake of CS-siRNA/PEITC&L-cRGD NPs via flow cytometry. As depicted in Fig. 4A, the mean fluorescence values for PBS, Free siRNA, CS-siRNA/PEITC&L NPs and CS-siRNA/PEITC&L-cRGD NPs were 66.81 ± 8.16, 68 ± 10.31, 3664 ± 312.43, and 5875.32 ± 514.59 respectively, in HUVEC cells. The mean fluorescence values for PBS, Free siRNA, CS-siRNA/PEITC&L NPs and CS-siRNA/PEITC&L-cRGD NPs were 67.43 ± 7.51, 74 ± 9.49, 6452 ± 417.47 and 11,618.36 ± 819.31 respectively, in A549 cells. There was no significant difference between the free siRNA group and the PBS group in HUVEC and A549 cells, indicating that the free siRNA, due to its cell membrane charge repulsion, resulted in an uptake efficiency of almost 0. The cellular uptake efficiencies of CS-siRNA/PEITC&L-cRGD NPs and CS-siRNA/PEITC&L NPs were significantly higher than that in the PBS group and the Free siRNA group. This indicated that the nanoparticles could significantly improve the cellular uptake of siRNA. And the cellular uptake efficiency of CS-siRNA/PEITC&L-cRGD NPs was 1.6-fold and 1.8-fold higher than that of CS-siRNA/PEITC&L NPs in HUVEC and A549 cells respectively, which indicated that the nanoparticles modified by cRGD improved the cellular uptake efficiency. This provides support for the intracellular uptake of nanoparticles in tumor tissues. In addition, in our previous study, CS-siRNA NPs were found to have high uptake efficiency in HUVEC and A549 cells, and after phospholipid wrapping, the surface charge was shifted to negative, which reduced the uptake efficiency. However, positively charged nanoparticles also face the risk of clearance by the phagocytic system and safety concerns such as hemolysis upon direct intravenous administration [34,35].

#### 3.2.4 Cellular endocytic mechanism

We further investigated the cellular endocytic mechanism using endocytosis inhibitors. As depicted in Fig. 4B, the most significant inhibitory effect was observed for HUVEC cells in the chlorpromazine group, exhibiting a mean fluorescence value of 1391.75 ± 526.89 (***P < 0.001) and an uptake inhibition rate of 73.01% ± 4.6%. No significant differences were detected between the indomethacin and amiloride groups in comparison to the control group. However, both groups still exhibited some degree of inhibition, with uptake inhibition rates of 27.15% ± 2.31% and 17.60% ± 3.21%, respectively. The group treated with β-cyclodextrin did not exhibit any inhibition of uptake. In the case of A549 cells, the amiloride group showed the most significant inhibitory effect, with a mean fluorescence value of 5642.89 ± 230.89 (**P < 0.01) and an uptake inhibition rate of 53.42% ± 3.82%. The group administered chlorpromazine subsequently recorded a mean fluorescence value of 8891.75 ± 416.89 (*P < 0.05) along with an uptake inhibition rate of 26.59% ± 3.12%. No uptake inhibition was observed in the indomethacin group and β-cyclodextrin group. Thus, the internalization of CS-siRNA/PEITC&L-cRGD NPs by HUVEC cells occurs predominantly through the clathrin and the caveolin pathways. For A549 cells, the CS-siRNA/PEITC&L-cRGD NPs are primarily internalized via the clathrin pathway and macropinocytosis.

#### 3.2.5 CLSM visualization

The distribution of nanoparticles within HUVEC and A549 cells was visualized using CLSM. As shown in Fig. 4C, red fluorescence signals of siRNA were observed in both cell types for the PBS(Control), Free siRNA, CS-siRNA/PEITC&L NPs, and CS-siRNA/PEITC&L-cRGD NPs groups. CS-siRNA/PEITC&L-cRGD NPs exhibited the highest level of Cy5 fluorescence, while Free siRNA showed almost no fluorescence signal. This is consistent with the result of the cellular uptake experiments, indicating that the cells were effectively able to uptake CS-siRNA/PEITC&L-cRGD NPs. Additionally, the co-delivery nanosystem can be effectively internalized by the cells, providing experimental support for further *in vivo* biological studies.

### 3.3 Tumor accumulation and tumor vascular system targeting

**Fig. 5:**
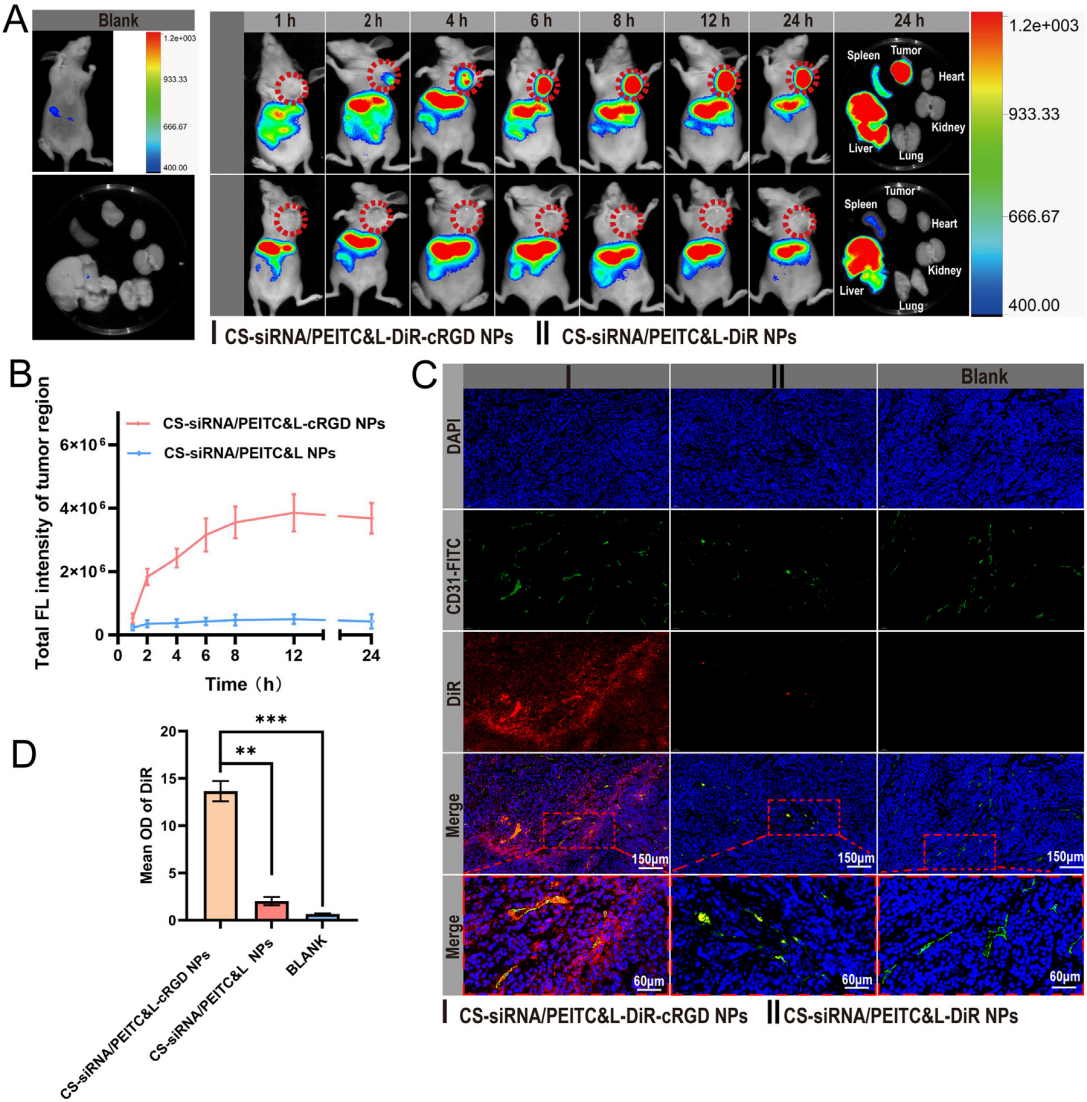
(A) *In vivo* fluorescence imaging of CS-siRNA/PEITC&L-DIR-cRGD NPs and CS-siRNA/PEITC&L-DIR NPs in BALB/c nude mice post tail vein injection (*n* = 3). (B) Graph representing total fluorescence intensity in the tumor region (*n* = 3). (C) Representative immunofluorescence images of tumor tissue sections prepared from paraffin. DAPI-stained nuclei are shown in blue, FITC—CD31-labeled tumor blood vessels in green, and CS-siRNA/PEITC&L-DIR-cRGD NPs and CS-siRNA/PEITC&L-DIR NPs in red. (D) Graph showing average fluorescence intensity of DIR (*n* = 3). Statistical significance is indicated as *P < 0.05, **P < 0.01, and ***P < 0.001.

To verify the nanoparticles' active tumor targeting ability and analyze their accumulation pattern, we injected equal molar amounts of cRGD-modified particles (CS-siRNA/PEITC&L-cRGD NPs) and unmodified particles (CS-siRNA/PEITC&L NPs) into the tail vein of female BALB/c nude mice once the average tumor volume reached 400 mm^3^. Both preparations' lipid membrane coatings were labeled using the near-infrared dye DiR for tracking *in vivo*. The results (Fig. 5A) showed that CS-siRNA/PEITC&L-DIR-cRGD NPs exhibited efficient tumor targeting ability compared to CS-siRNA/PEITC&L-DIR NPs. And fluorescent signals from the liver and spleen were observed in Fig. 5A. This suggests that macrophages phagocytose some of the nanoparticles because the liver and spleen are part of the mononuclear phagocyte system [36]. The pattern of accumulation was confirmed by quantitative analysis of the mean fluorescence intensity in the tumor area (Fig. 5B). At 2 h after administration, the CS-siRNA/PEITC&L-DIR-cRGD NPs group exhibited a significant accumulation difference compared to the unmodified cRGD group, with a 5.29-fold higher mean fluorescence intensity in the tumor region. At the 8-h mark, the CS-siRNA/PEITC&L-DIR-cRGD NPs group reached the accumulation plateau, suggesting efficient accumulation of targeted agents in the tumor tissue. The difference in tumor accumulation between CS-siRNA/PEITC&L-DIR-cRGD NPs and CS-siRNA/PEITC&L-DIR NPs increased over time. At 8 h after administration, the mean fluorescence intensity of the former group was 7.68 times higher than that of the latter group (Fig. 5B). Imaging results for both *in vivo* and isolated tumors (Fig. 5A and C) indicated that the group treated with CS-siRNA/PEITC&L-DIR-cRGD NPs maintained effective fluorescence distribution in the tumor region 24 h post-administration. This provides direct evidence of the extended accumulation of CS-siRNA/PEITC&L-DIR-cRGD NPs within tumors. In contrast, CS-siRNA/PEITC&L-DIR NPs circulated only in the blood and were not effectively retained in the tumor region.

CS-siRNA/PEITC&L-DIR-cRGD NPs could efficiently bind to integrin α_v_β_3_ on the surface of tumor vascular endothelial cells and tumor cells due to the modification of cRGD on the lipid membrane surface. To further verify the targeting ability of CS-siRNA/PEITC&L-DIR-cRGD NPs to the tumor vasculature, isolated tumors were processed into paraffin sections and examined by immunofluorescence. FITC—CD31 antibody (green) was used to label tumor vessels. The results (Fig. 5C) showed the distribution of DiR (red) in the tumor tissue 24 h after administration. The group treated with CS-siRNA/PEITC&L-DIR-cRGD NPs illustrated proficient fluorescence distribution in the tumor tissue, consistent with *in vivo* imaging (Fig. 5A). In all fields of view (Fig. 5C), the DiR group within the CS-siRNA/PEITC & l-DIR-cRGD NPs showed a fluorescence distribution pattern that centered on tumor vessels and spread in all directions. This indicated the selectivity of CS-siRNA/PEITC&L-DIR-cRGD NPs for active targeting of tumor endothelial vessels and their ability to penetrate into tumor tissues. The quantitative analysis of the mean fluorescence intensity of tumor tissue sections (Fig. 5D) displayed the same results. The CS-siRNA/PEITC&L-DIR-cRGD NPs group showed significant differences from the unmodified cRGD group, with the mean fluorescence intensity of tumor tissue sections being 6.74-fold higher than that of the unmodified cRGD group. The CS-siRNA/PEITC&L-DIR-cRGD NPs group exhibited proficient tumor targeting and penetrating abilities, serving as the foundation for the subsequent drug effectiveness of the preparation.

### 3.4 *In vivo* antitumor efficacy

#### 3.4.1 Tumor growth inhibition

**Fig. 6:**
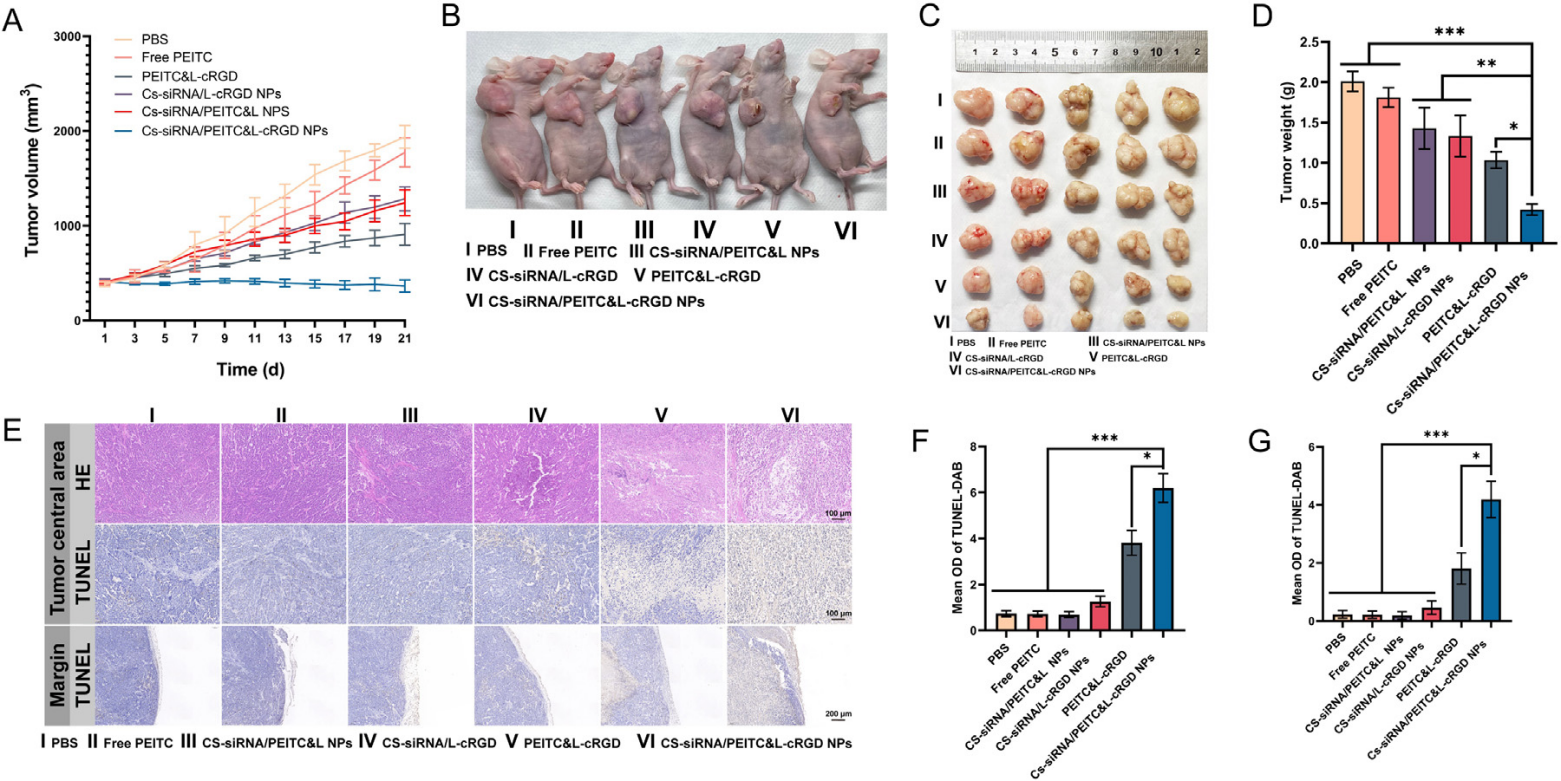
(A) Time-dependent changes in tumor volume for different treatment groups (*n* = 5). (B) Representative images of mice from each treatment group at the end of treatment. (C) Images of excised tumor tissues from each treatment group after 21 d of treatment. (D) Weights of the excised tumors from each group (*n* = 5). (E) Representative images of tumor sections stained with HE and TUNEL after treatment, embedded in paraffin (*n* = 3). (F-G) Graph showing the mean optical density (OD) of TUNEL-DAB (F) in the central region of tumors and (G) in the marginal region of tumors (*n* = 5). Statistical significance is indicated as *P < 0.05, **P < 0.01, and ***P < 0.001.

In established BALB/c nude tumor-bearing mice models, we validated the ability of CS-siRNA/PEITC&L NPs to target and infiltrate tumors. To further verify the anti-tumor efficacy of CS-siRNA/PEITC&L NPs, equal molar amounts of CS-siRNA/PEITC&L-cRGD NPs and control groups (PBS, free PEITC, CS-siRNA/PEITC&L NPs, CS-siRNA/ l-cRGD NPs, and PEITC&L-cRGD) were injected into the tail vein of BALB/c nude mice when the tumor volume grew to 400 mm^3^. The tumor volume change curve (Fig. 6A) reveals that the CS-siRNA/PEITC&L-cRGD NPs group maintained the initial volume after the first treatment, indicating efficient suppression of tumor growth. The tumor growth inhibition rate can be calculated from the tumor volume. On Day 11 after treatment, the CS-siRNA/PEITC&L-cRGD NPs group exhibited a tumor growth inhibition value of 63.85%, which was significantly higher compared to the CS-siRNA/PEITC&L NPs group (25.32%, ***P < 0.001), CS-siRNA/L-cRGD NPs group (27.63%, ***P < 0.001), and PEITC&L-cRGD group (42.32%, **P < 0.05). On Day 21 of treatment, the tumor growth inhibition rate was significantly higher in the CS-siRNA/PEITC&L-cRGD NPs group (81.19%) compared to the CS-siRNA/PEITC&L NPs group (35.96%, ***P < 0.001), CS-siRNA/L-cRGD NPs group (33.83%, ***P < 0.001), and PEITC&L-cRGD group (53.10%, ***P < 0.001). The group administered with free PEITC did not exhibit statistically significant suppression of the tumor, indicating that free PEITC was unsuccessful in inhibiting tumor growth effectively due to random tissue distribution. The CS-siRNA/PEITC&L-cRGD NPs group exhibited efficient anti-tumor effects and effectively inhibited tumor growth and proliferation. Fig. 6B displays representative images of mice in each group at the treatment endpoint, and tumors in the CS-siRNA/PEITC&L-cRGD NPs group were significantly different in appearance and size from those in each group. Fig. 6C displays the images of the tumors isolated from each group, revealing notable variations in their sizes in comparison to the CS-siRNA/PEITC&L-cRGD NPs group. Additionally, there are substantial differences in the tumor weight (Fig. 6D) amongst each group. The above results indicate that CS-siRNA/PEITC&L-cRGD NPs possess efficient anti-tumor ability and can effectively inhibit tumor growth and proliferation.

#### 3.4.2 Tumor necrosis and margin inhibition

To further verify the pro-tumor necrosis and margin suppression ability of CS-siRNA/PEITC&L-cRGD NPs, paraffin-embedded sections of isolated tumors were stained by HE and TUNEL immunohistochemistry. The results showed (Fig. 6E) that the CS-siRNA/PEITC&L-cRGD NPs group had a significant pro-apoptotic necrotic effect on the tumor center and margin. The apoptosis rate can be calculated from the mean optical density bar graph by semi-quantitative analysis of TUNEL-positive areas by optical density. The results of apoptosis in the central tumor region showed (Fig. 6F) that the apoptosis rate of CS-siRNA/PEITC&L-cRGD NPs group on tumor cells was 94.51%, which was significantly higher than that of PEITC&L-cRGD group (50.40%, **P < 0.01). The groups treated with free PEITC, CS-siRNA/PEITC&L NPs, and CS-siRNA/L-cRGD demonstrated no significant pro-apoptotic effects on tumor cells according to statistical analysis. The results of apoptosis in the tumor margin (Fig. 6G) indicated that the CS-siRNA/PEITC&L-cRGD NPs group had a significantly higher apoptosis rate on tumor cells (88.48%) compared to the PEITC&L-cRGD group (37.70%, ***P < 0.001). Similarly, the groups treated with free PEITC, CS-siRNA/PEITC&L NPs, and CS-siRNA/L-cRGD did not show statistically significant pro-apoptotic effects on tumor margin cells.

The group treated with CS-siRNA/PEITC&L-cRGD NPs demonstrated effective suppression of tumor growth and inhibited tumor margin survival. It has been shown that siRNA and PEITC can effectively kill tumor cells and prevent marginal recurrence by being widely distributed within the tumor. Although siRNA does not possess pro-apoptotic necrotic ability, its co-delivery with PEITC can effectively inhibit tumor angiogenesis, leading to decreased nutrient supply and subsequently enhanced tumor necrosis.

### 3.5 Anti-tumor angiogenesis validation

#### 3.5.1 Immunohistochemical analysis of tumor vascular inhibition

**Fig. 7:**
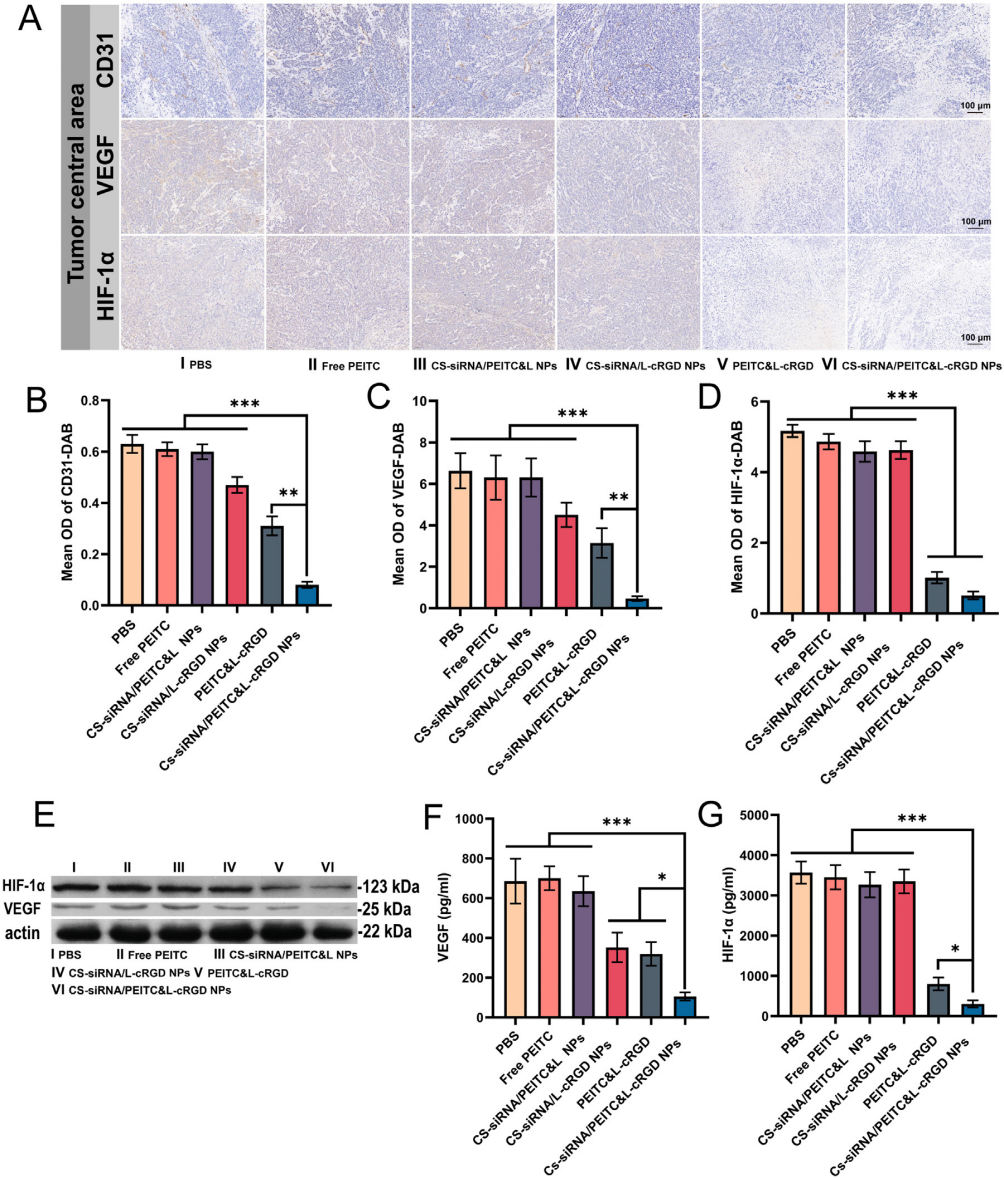
(A) Representative images of paraffin-embedded tumor marginal area sections stained with CD31-DAB, VEGF, and HIF-1α after treatment. (B-D) Mean OD of (B) CD31-DAB, (C) VEGF-DAB and (D) HIF-1α-DAB in the marginal region of tumors (*n* = 5). (E) Western blot analysis depicting the expression of VEGF and HIF-1α proteins across different treatment groups. (F-G) ELISA results representing the expression of (F) VEGF protein and (G) HIF-1α protein across different treatment groups (*n* = 3). Statistical significance is indicated as *P < 0.05, **P < 0.01, and ***P < 0.001.

According to Fig. 7A, there was no significant difference observed in the CD31-DAB positive regions between the free PEITC and CS-siRNA/PEITC&L NPs groups and the PBS group. This suggests that the free PEITC and CS-siRNA/PEITC&L NPs groups had a comparatively weaker capability to inhibit the tumor vasculature. The positive area was slightly reduced in the CS-siRNA/L-cRGD group, demonstrating some inhibitory effect on tumor vasculature. The positive regions exhibited a significant reduction in the PEITC&L-cRGD group, which serves as evidence of the tumor vasculature inhibitory effect through targeted PEITC delivery. Almost no areas of positive effect were observed in the group receiving CS-siRNA/PEITC&L-cRGD NPs, clearly indicating the significant inhibitory impact of the co-delivery system on tumor vasculature.

The CD31-DAB positive region was analyzed using optical density (OD) to measure it semi-quantitatively, and the inhibition rate was calculated from the mean optical density bar graph. The results showed (Fig. 7B) that the inhibition rate of CD-31-DAB in the CS-siRNA/PEITC&L-cRGD NPs group was 87.30%, which was significantly higher than that in the CS-siRNA/L-cRGD group (25.40%, ***P < 0.001) and PEITC&L-cRGD group (50.79%, **P < 0.01). The inhibition rates of the free PEITC group and CS-siRNA/PEITC&L NPs group did not show statistical significance. The results of CD31-DAB immunohistochemistry showed that the single drug treatment groups did not achieve the desired tumor vascular inhibition effect, while the co-delivery treatment group exhibited efficient tumor vascular inhibition. This demonstrates that the combined delivery strategy of PEITC and siRNA effectively inhibited tumor vascularization. Evaluation of anti-tumor potency and anti-tumor neovascularization showed that he dual mechanism of action of anti-tumor angiogenesis and pro-tumor apoptosis has achieved efficient tumor vasculature inhibition and pro-tumor apoptosis.

#### 3.5.2 Immunohistochemical analysis of VEGF and HIF-1α inhibition

Tumor angiogenic capacity is influenced by VEGF and HIF-1α levels, which are critical factors in tumor angiogenesis. To further validate the mechanism behind the inhibition of tumor angiogenesis by the CS-siRNA/PEITC&L-cRGD NPs group, we performed an analysis of VEGF and HIF-1α expression using immunohistochemical VEGF-DAB and HIF-1α-DAB staining. As shown in Fig. 7A, the group treated with CS-siRNA/PEITC&L-cRGD NPs exhibited significant inhibition of VEGF protein expression. The inhibitory potency of VEGF expression showed similarity in the PEITC&L-cRGD and CS-siRNA/L-cRGD NPs groups, whereas the CS-siRNA/PEITC&L-cRGD NPs group demonstrated the strongest inhibitory potency. On the other hand, the free PEITC and CS-siRNA/PEITC&L NPs groups displayed no significant inhibitory effect The group of CS-siRNA/PEITC&L-cRGD NPs was successful in suppressing HIF-1α protein expression, whereas the groups of free PEITC, CS-siRNA/L-cRGD NPs, and CS-siRNA/PEITC&L NPs did not demonstrate any significant inhibitory effect on HIF-1α. The regions positive for VEGF-DAB and HIF-1α-DAB were analyzed using semi-quantitative measures of optical density. The inhibition rate was calculated based on the mean optical density bar graph. The results of VEGF (Fig. 7C) demonstrated effective VEGF expression inhibition by the CS-siRNA/PEITC&L-cRGD NPs group, displaying an inhibition rate of 92.91%. This was significantly greater than the CS-siRNA/L-cRGD (31.97%, ***P < 0.001) and PEITC&L-cRGD NPs (52.48%, **P < 0.01) groups. The results for HIF-1α (Fig. 7D) indicate that the group received CS-siRNA/PEITC&L-cRGD NPs demonstrated a 90.13% inhibition rate, efficiently suppressing HIF-1α expression. This was significantly higher than the PEITC&L-cRGD group (75.78%, *P < 0.05). The immunohistochemical staining results presented provide protein-level evidence that the group treated with CS-siRNA/PEITC&L-cRGD NPs effectively inhibits the expression of VEGF and HIF-1α, thus elucidating the mechanism underlying the co-delivery system's anti-tumor angiogenesis effects.

#### 3.5.3 Western blot and ELISA analysis

To further verify the inhibitory effect of CS-siRNA/PEITC&L-cRGD nanoparticles on the expression of VEGF and HIF-1α, we used the Western blot technique to detect differences in HIF-1α and VEGF protein expression in tumor tissues at the end of treatment for each group. The results revealed (Fig. 7E) an efficient inhibition of VEGF and HIF-1α protein expression by the CS-siRNA/PEITC&L-cRGD NPs treatment. Similar inhibition of VEGF expression was observed in both the PEITC&L-cRGD and CS-siRNA/L-cRGD NPs groups, with the most pronounced inhibition observed in the CS-siRNA/PEITC&L-cRGD NPs group. The groups administered with free PEITC and CS-siRNA/PEITC&L NPs did not exhibit any significant inhibitory effects. HIF-1α expression was mitigated by PEITC&L-cRGD, while the free PEITC group, CS-siRNA/L-cRGD NPs group, and CS-siRNA/PEITC&L NPs group did not noticeably affect HIF-1α.

The ELISA method was used to measure the varying levels of VEGF and HIF-1α proteins within the tumor tissues of every group. The VEGF quantification results (Fig. 7F) indicated that the CS-siRNA/PEITC&L-cRGD NPs group effectively inhibited VEGF expression, with an inhibition rate of 84.63%. This rate was significantly higher than that of the CS-siRNA/L-cRGD group (48.60%, **P < 0.01) and the PEITC&L-cRGD NPs group (53.41%, **P < 0.01). No statistically significant inhibition of VEGF was observed in the free PEITC and CS-siRNA/PEITC&L NPs groups. The HIF-1α quantification analysis (Fig. 7G) demonstrated that the CS-siRNA/PEITC&L-cRGD NPs group efficiently inhibited HIF-1α expression with an inhibition rate of 91.51%, which was significantly higher than that of the PEITC&L-cRGD group (77.52%, *P < 0.05). CS-siRNA/PEITC&L-cRGD NPs group exhibited a higher inhibitory effect in comparison to the PEITC&L-cRGD group. This result can be attributed to the combined effects of siRNA and PEITC in preventing tumor angiogenesis through several pathways that trigger tumor dysfunction or necrosis. As a result, the expression of HIF-1α is further reduced. No significant inhibitory effects on HIF-1α were observed in the group treated with free PEITC, the CS-siRNA/PEITC&L NPs group, or the CS-siRNA/L-cRGD NPs group. The results of the VEGF and HIF-1α assays indicate that the CS-siRNA/PEITC&L-cRGD NPs group effectively suppressed the expression of VEGF and HIF-1α, indicating that the PEITC and siRNA-targeted co-delivery approach has the potential to inhibit tumor angiogenesis. Additionally, the inhibitory effects of both groups of CS-siRNA/L-cRGD NPs and PEITC&L-cRGD NPs were weaker compared to the effects of CS-siRNA/PEITC&L-cRGD NPs. This result provides direct evidence of the synergistic effect of PEITC and siRNA through their precise co-delivery. In comparison with single-target therapy, CS-siRNA/PEITC&L-cRGD NPs effectively inhibited tumor neovascularization and enhanced tumor necrosis by blocking nutrient supply. For the first time, we verified that the simultaneous suppression of VEGF and HIF-1α is critical for antiangiogenic therapy and can more efficiently halt tumor vasculature, which may help advance antiangiogenic research by inhibiting multiple targets.

### 3.6 Preliminary safety evaluation

**Fig. 8:**
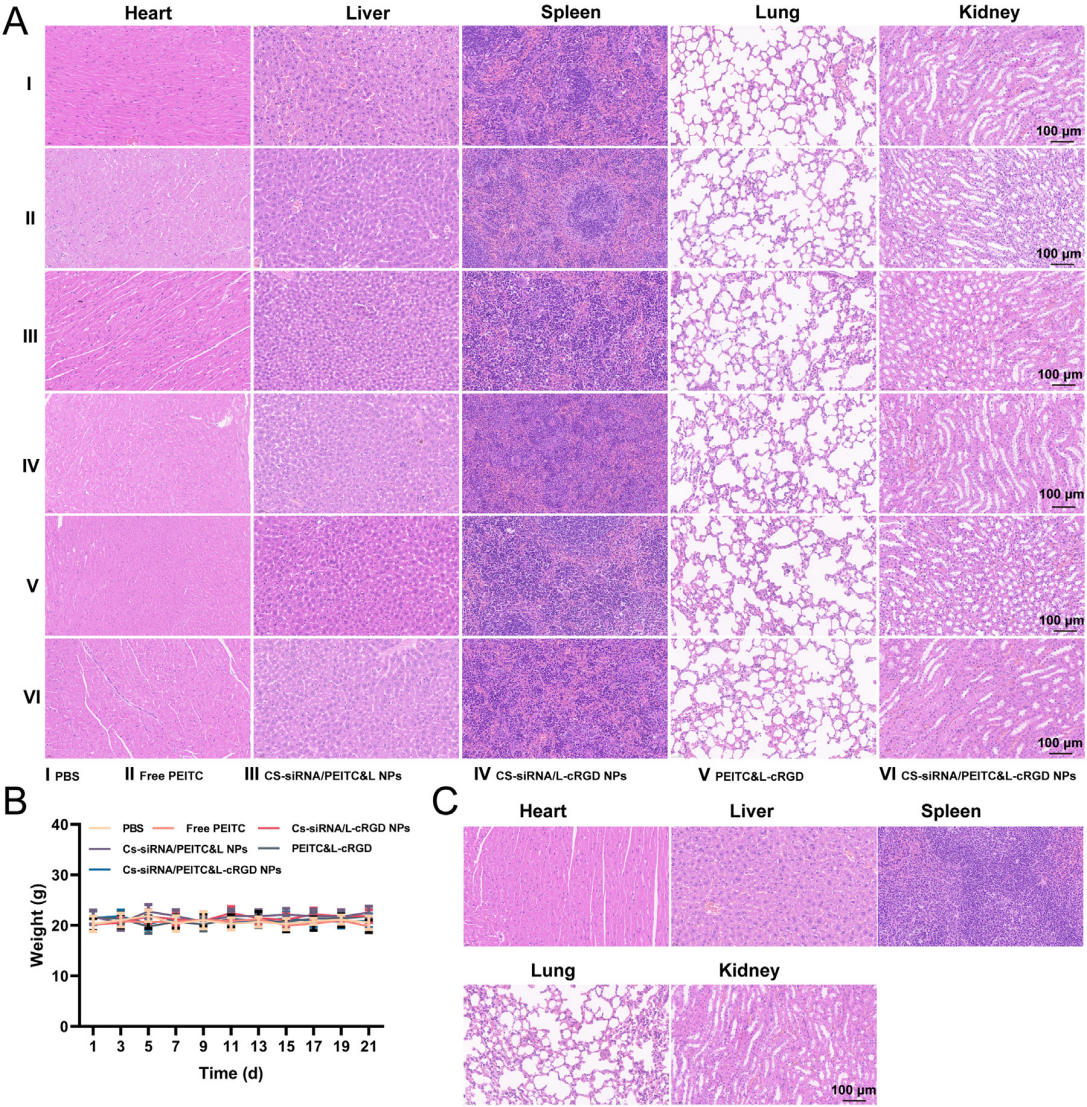
(A) Representative images of HE-stained sections from various vital organs (heart, liver, spleen, lung, kidney) in mice, following 21 d of anti-tumor treatment (*n* = 5). No conspicuous signs of tissue damage or ectopic thrombus were observed. (B) Graph illustrating changes in the body weight of mice during the course of anti-tumor treatment (*n* = 5). The body weight of mice across all groups remained largely stable. (C) Representative HE-stained images of vital organs (heart, liver, spleen, lung, and kidney) from healthy BALB/c nude mice subjected to high-dose (three times the regular dose) tolerance experiments (*n* = 5). Tissues from all examined organs displayed no abnormal damage.

To evaluate the safety of CS-siRNA/PEITC&L-cRGD NPs, we logged the changes in the body weight of mice during treatment, monitored their physiological condition, and examined their major organs histologically. Histological examination (Fig. 8A) revealed no significant cell necrosis in the vital organs of the treated mice. No significant differences were observed in the physiological status of organs between the treated group and healthy mice, and there were no treatment-related acute toxic events detected. Specifically, nanoparticles accumulate not only in tumor tissue but also in the liver. In HE staining of the liver, the nanoparticles did not cause histopathological changes. The hepatocytes were well-arranged and structured, with no infiltration of monocytes, no proliferation of Kupffer cells, no hepatocyte swelling or vacuolar degeneration, and no focal deformations, focal necrosis generation, or fibrosis development. There was no significant change in body weight (Fig. 8B), suggesting that the mice maintained good physical condition throughout the duration of the treatment. No signs of respiratory distress, asymmetric movements, or sudden deaths were detected in any of the groups. In high-dose tolerance experiments using triple doses of nanoparticles, there was no significant decrease in survival or organ damage observed in healthy mice, when compared to the previous experiments. These results suggested that the CS-siRNA/PEITC&L-cRGD NPs were well tolerated (Fig. 8C).

The preliminary safety evaluation demonstrated that CS-siRNA/PEITC&L-cRGD NPs exhibit outstanding biocompatibility and can induce potent anti-tumor effects while maintaining safety. The safety of CS-siRNA/PEITC&L-cRGD NPs as a therapy depends on two primary factors. First, CS-siRNA/ PEITC & l-cRGD NPs are capable of preserving the structural stability of the preparation. Phospholipids could effectively encapsulate the inner-shell CS-siRNA NPs, while PEITC could be stably loaded into the phospholipid bilayer, thus improving the bioavailability of CS-siRNA NPs and PEITC. Secondly, the surface of the cell membrane is negatively charged, it has a repulsive effect on the nanoparticles, which are also negatively charged. The negative surface charge of CS-siRNA/PEITC&L-cRGD NPs reduces uptake by non-targeted cells, thereby decreasing toxic effects. In addition, the modification of cRGD exhibited exceptional tumor-targeting abilities, resulting in reduced uptake by non-targeted cells.

## 4 Conclusion

In conclusion, this study demonstrated that CS-siRNA/PEITC&L-cRGD NPs could effectively accumulate in tumor tissues, efficiently inhibit the accumulation of VEGF and HIF-1α expression, effectively curb tumor angiogenesis, block nutrient supply, and promote tumor apoptosis. This provided a novel therapeutic strategy for clinical anti-angiogenic therapy by utilizing the dual mechanisms of inhibition of tumor angiogenesis and promotion of tumor cell apoptosis, which will promote multi-targeted anti-tumor angiogenesis and pro-apoptosis research. The co-delivery nanosystems, equipped with a modifiable, drug-carrying lipid coating and an inner shell with efficient gene-drug loading capability, present themselves as promising candidates for chemotherapy and gene-drug combination therapeutic strategies.

## Conflicts of interest

The authors declare no conflict of interest.

## Appendix Supplementary materials

Image, application 1
