# Supplementary material for: Targeted anti-cancer therapy: Co-delivery of VEGF siRNA and Phenethyl isothiocyanate (PEITC) via cRGD-modified lipid nanoparticles for enhanced anti-angiogenic efficacy
Source: Asian J Pharm Sci. 2024 Feb 23;19(2):100891. doi: 10.1016/j.ajps.2024.100891 (PMC10990863; doi:10.1016/j.ajps.2024.100891)
Supplement: Supplementary file 1 [file mmc1.docx]

**Supplementary Material**

**Targeted Anti-Cancer Therapy: Co-Delivery of VEGF siRNA and Phenethyl isothiocyanate (PEITC) via cRGD-Modified Lipid Nanoparticles for Enhanced Anti-Angiogenic Efficacy**

**1. Loading capability of CS NPs for siRNA**

The loading capability of chitosan nanoparticles (CS NPs) for siRNA was assessed using agarose gel electrophoresis. CS-siRNA NPs were synthesized by allowing CS NPs to interact with siRNA at various mass ratios (1:1, 2:1, 3:1, 5:1, 7:1, and 10:1) for 20 min at room temperature. Free siRNA served as the control. As indicated in S1, migration bands of siRNA were clearly visible for the free siRNA group. However, these migration bands gradually disappeared as siRNA was incorporated with chitosan at increasing mass ratios (1:1, 2:1, 3:1, 5:1, 7:1, and 10:1). The siRNA migration bands completely vanished when the mass ratio reached 10:1, signifying that CS NPs can effectively bind to negatively charged siRNAs via electrostatic interactions, thus forming stable complexes. This confirms the efficient loading capacity of CS NPs for siRNAs.


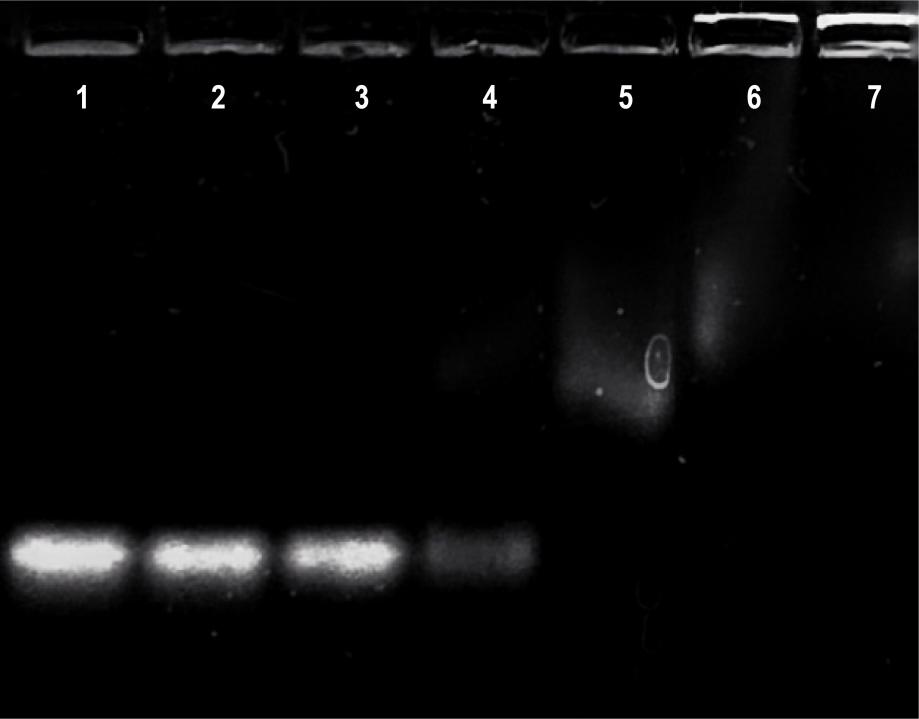


S1. Agarose gel retardation assay illustrating the binding capability of siRNA to CS NPs at various CS-to-siRNA weight ratios. Lanes are as follows: Lane 1 - Free siRNA; Lanes 2 to 7 - CS-siRNA NPs at weight ratios of 1:1, 2:1, 3:1, 5:1, 7:1, and 10:1 respectively. Each experiment was conducted in triplicate (*n* = 3).

**2.** **Optimization of the coating effect of CS-siRNA/PEITC&L NPs**

S2. illustrates the progressive enlargement of the particle size of CS-siRNA/PEITC&L NPs as the phospholipid ratio increases. Specifically, when the mass ratio of phospholipid to chitosan escalated from 0 to 2:1, the particle size of CS-siRNA/PEITC&L NPs grew from approximately 125.2 nm to around 149.9 nm. Concurrently, the zeta potential declined from approximately 24.81 mV to -7.21 mV. This suggests that at a phospholipid to chitosan mass ratio of 2:1, the positively charged CS-siRNA NPs can be entirely enveloped by the negatively charged phospholipids. This results in the reversal of the surface charge of CS-siRNA NPs, creating a negatively charged phospholipid surface. Given that CS-siRNA NPs can be fully encapsulated by phospholipids at the smallest particle size at this ratio, it is considered to be the optimal ratio.


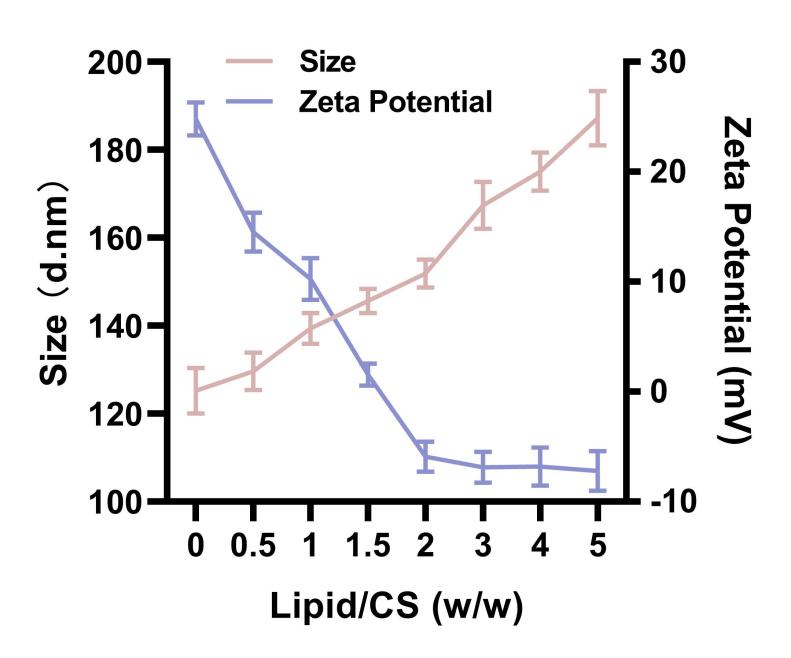


S2. Particle size and zeta potential of CS-siRNA/PEITC&L NPs at different lipid-to-CS weight ratios (w/w). Each condition was tested in triplicate (*n* = 3).

1. **The UV spectra of PEITC**


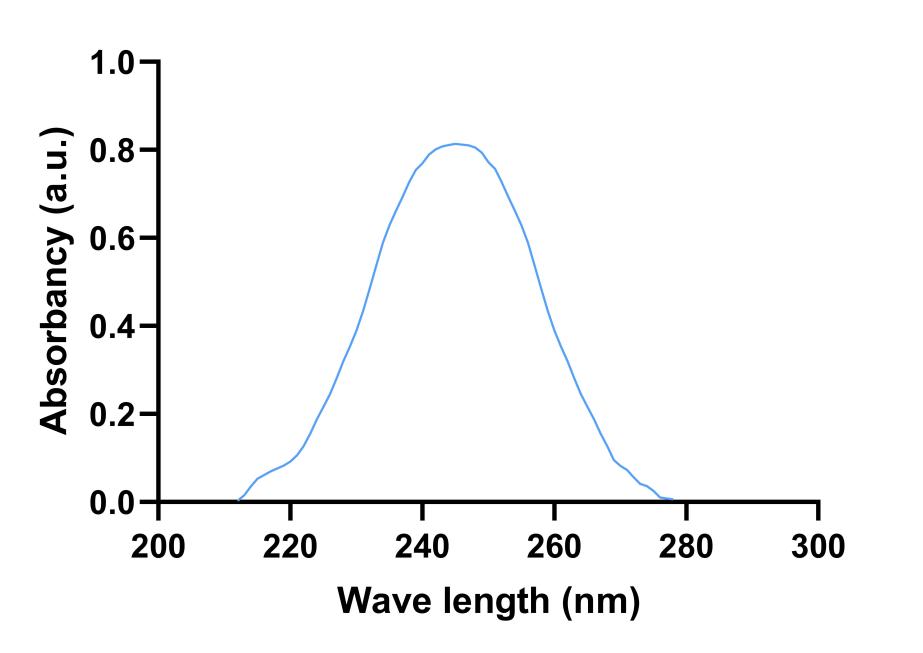


S3. The UV spectra of PEITC

1. ***In vitro* silencing efficiency of siRNA**

*In vitro* silencing efficiency of siRNA was evaluated using HUVEC and A549 cells. Both cell types (at a density of 3×10⁵ cells per well) were separately seeded into 6-well plates, respectively, and anoxic cultured overnight. Cells were then treated with CS-siRNA/L-cRGD NPs using concentrations that corresponded to final siRNA concentrations of 0, 10, 20, 30, and 50 nM. VEGF protein expression was analyzed using Western Blot. As shown in S4, siRNA concentration-dependently inhibited VEGF protein expression. This result demonstrated that the siRNA sequence has the ability to silence VEGF protein expression.

**
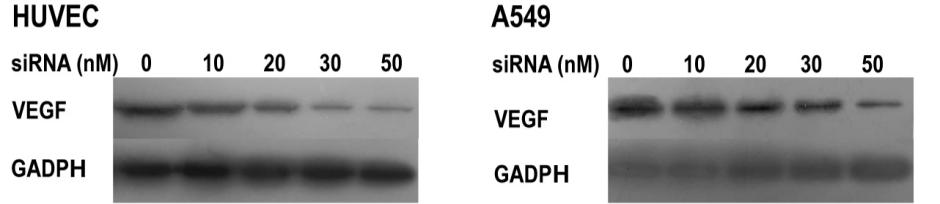
**

S4. Silencing efficiency of siRNA on HUVEC cells and A549 cells.
